# Relationship between blood-cerebrospinal fluid barrier integrity, cardiometabolic, and inflammatory factors in schizophrenia-spectrum disorders

**DOI:** 10.1016/j.bbih.2025.101024

**Published:** 2025-05-27

**Authors:** Vladislav Yakimov, Iris Jäger, Lukas Roell, Emanuel Boudriot, Verena Meisinger, Mattia Campana, Lenka Krčmář, Sean Halstead, Nicola Warren, Dan Siskind, Isabel Maurus, Alkomiet Hasan, Peter Falkai, Andrea Schmitt, Florian J. Raabe, Daniel Keeser, CDP-Working Group, Elias Wagner, Joanna Moussiopoulou

**Affiliations:** a Department of Psychiatry and Psychotherapy, LMU University Hospital, LMU Munich, Munich, Germany; b International Max Planck Research School for Translational Psychiatry (IMPRS-TP), 80804, Munich, Germany; c NeuroImaging Core Unit Munich (NICUM), LMU University Hospital, LMU Munich, 80336, Munich, Germany; d Max Planck Institute of Psychiatry, 80804, Munich, Germany; e Department of Psychiatry and Psychotherapy, Medical Faculty, LVR Hospital of the Heinrich Heine University Düsseldorf, Düsseldorf, Germany; f Medical School, The University of Queensland, Brisbane, QLD, Australia; g Metro South Addiction and Mental Health, Brisbane, QLD, Australia; h Department of Psychiatry, Psychotherapy and Psychosomatics, Faculty of Medicine, University of Augsburg, 86156, Augsburg, Germany; i German Center for Mental Health (DZPG), Partner Site Munich, Augsburg, Germany; j Laboratory of Neuroscience (LIM27), Institute of Psychiatry, University of Sao Paulo, São Paulo, Brazil; k Evidence-based Psychiatry and Psychotherapy, Faculty of Medicine, University of Augsburg, Stenglinstrasse 2, 86156, Augsburg, Germany

**Keywords:** Psychosis, CNS barriers, Cardiovascular health, Immunity, Cerebroventricular system

## Abstract

The blood-cerebrospinal fluid barrier (BCB) is impaired in a substantial proportion of individuals with schizophrenia-spectrum disorders (SSD). Even though disruption of the BCB is associated with higher symptom severity, factors linked to BCB disruption in SSDs have been minimally investigated.

To address this gap, 57 inpatients with SSD underwent cerebrospinal fluid (CSF), blood analyses, and comprehensive clinical assessments. In a subgroup of 28 participants, structural magnetic resonance imaging (MRI) was performed. We developed a BCB dysfunction score, employing principal component analysis of CSF/serum albumin, CSF/serum IgG ratios, and total protein levels in CSF, with higher values indicating stronger abnormalities. Bayesian linear and logistic regression models were calculated to explore the associations between BCB integrity and cardiometabolic, inflammatory, cerebroventricular, and clinical measures.

Our results indicated very strong evidence for a negative association between the BCB dysfunction score and high-density lipoprotein cholesterol, as well as extreme evidence for positive associations between the BCB dysfunction score and total, low-density lipoprotein cholesterol, and triglycerides. Furthermore, there was moderate evidence of a positive association between BCB dysfunction score and treatment resistance. We did not find evidence of associations between the BCB composite score and any other assessed cardiometabolic, inflammatory, or cerebroventricular measures.

These findings suggest that BCB integrity is associated with dyslipidemia and treatment resistance in SSD, highlighting the interplay between cardiometabolic risk factors and brain health in SSD. Addressing cardiometabolic health in individuals with SSD could influence the integrity of the BCB and, consequently, clinical trajectories.

## 1 Introduction

Schizophrenia-spectrum disorders (SSD) are among the leading causes of morbidity worldwide due to high rates of treatment resistance as well as cognitive and functional impairment (Owen et al., 2016). Compared to the general population, life expectancy is reduced by ∼15 years (Hjorthøj et al., 2017), not only due to high rates of suicide but also due to the high prevalence of somatic comorbidities such as cardiovascular diseases (Pedersen et al., 2018). Of note, data from drug-naïve first-episode schizophrenia (SCZ) patients (Sayed et al., 2023; Bioque et al., 2018) as well as genetic studies (Rødevand et al., 2023; Xiao et al., 2024) indicate that metabolic disturbances are not just sequelae of unhealthy lifestyle factors and adverse drug reactions but potentially contribute to SSD pathophysiology.

Metabolic syndrome (MetS) is defined as the occurrence of at least three interrelated cardiometabolic risk factors, including central obesity, hypertension, hyperglycemia, and dyslipidemia (Alberti et al., 2009). It is highly prevalent in SSD (Mitchell et al., 2013), and high-quality meta-analytic evidence suggests a link between MetS and cognitive impairment in people with SCZ (Hagi et al., 2021; Bora et al., 2017). Furthermore, a study from the ENIGMA Working Group demonstrated that body-mass-index (BMI) (as a proxy for obesity) was additively associated with structural alterations in many of the same brain regions affected in schizophrenia, including changes in cortical thickness (McWhinney et al., 2022). Despite the limitations of the cross-sectional nature of this data, they suggest a complex relationship between metabolic disturbances, brain structure, and cognitive functions in individuals with SSD.

Even though the exact pathophysiology of SSD remains elusive, recent research has highlighted the role of immune system dysfunction as a contributing factor in a subset of individuals with SSD (Halstead et al., 2023; Bishop et al., 2022). Converging evidence from genetic (Trubetskoy et al., 2022), as well as large-scale epidemiological studies (Benros et al., 2011, 2014) points to the role of immune dysregulation in SSD (The blood, 2018). Furthermore, preliminary evidence from multimodal studies combining neuroimaging data with peripheral inflammatory markers suggests a link between peripheral low-grade inflammation and structural, as well as functional cerebral changes, which potentially increase the risk for more pronounced psychopathology (Bishop et al., 2022).

Impairments of the blood-brain barrier (BBB) (Moussiopoulou et al., 2025) and the blood-cerebrospinal fluid (CSF) barrier (BCB) (Campana et al., 2022; Rømer et al., 2023) are common findings in SSD. While both barriers share similar functions, the BBB is spread throughout the brain, whereas the BCB is mainly formed by epithelial cells of the choroid plexus (ChP) and the arachnoid membrane facing the CSF (Yakimov et al., 2023; Tumani et al., 2017). Of note, CSF/serum ratios of proteins such as albumin and immunoglobulin G (IgG) are technically an indirect measure of BCB integrity, rather than BBB integrity, despite frequent misinterpretation in the literature (Yakimov et al., 2023; Tumani et al., 2017). Some of the functions of both interfaces include ensuring a stable milieu (The blood, 2018), which is crucial for intact neural signaling in the brain, and transporting nutrients, oxygen, and waste products (The blood, 2018). BCB and BBB also act as central immunological nodes, building the interface between central and peripheral immune system and coordinating access of leukocytes to the central nervous system (CNS) (Pollak et al., 2018; Obermeier et al., 2013). Hence, a barrier disturbance is likely to be associated with disruption of brain homeostasis and functioning, with potential relevance for SSD (psycho)pathology (Pollak et al., 2018). Despite shared functions between BBB and BCB, much of the existing literature on barrier dysfunction in psychiatric disorders has predominantly focused on the BBB. In contrast, the BCB has received comparatively little attention. However, a growing body of evidence points to alterations of the ChP in SSD, including increased morphological variability (Yakimov et al., 2024), altered ChP epithelia (Williams et al., 2023), and upregulation of immune-related genes in the ChP (Kim et al., 2016). Despite the anatomical and functional relevance of the ChP for BCB (Obermeier et al., 2013) and the accumulating evidence for alterations in both ChP (Lizano et al., 2019) and BCB (Campana et al., 2022) in SSD, the relationship between these factors has not yet been systematically investigated.

Although the exact cause of BCB disruption in SSD is unknown, one hypothesis states that it occurs following a primary inflammatory insult (Pollak et al., 2018). Once disrupted, the brain might be susceptible to peripheral immune effectors, which can disturb brain function (Pollak et al., 2018). Since blood vessels and especially endothelial cells are an integral building block of the BCB, vascular dysfunction, as a result of a high cardiometabolic burden (e.g., hypertension, diabetes, obesity, and dyslipidemia), might also be relevant.

To address these questions, in our study, we 1) explored the associations between BCB integrity and different disease characteristics (duration of illness, duration of antipsychotic treatment, first episode psychosis status) and clinical factors (global functioning, treatment resistance, positive, negative, and general symptoms, cognitive impairment). Next, we 2) studied the links between BCB integrity and peripheral inflammatory markers as well as 3) cardiometabolic risk factors. Lastly, in a subgroup of participants with SSD, we 4) investigated the associations between BCB integrity and volumes of cerebroventricular regions, such as choroid plexus and lateral ventricles.

## 2 Methods

### 2.1 Participants

This study was conducted in the context of the IMPACT study (Moussiopoulou et al., 2025), an ongoing add-on study to the Munich Mental Health Biobank (project numbers 18–716 and 21–0183) (Kalman et al., 2022), and approved by the ethics committee of the Faculty of Medicine, LMU University Hospital Munich (project number 21–1139).

The recruitment of study participants was conducted at the Department of Psychiatry and Psychotherapy, LMU University Hospital, LMU Munich, Germany, between July 26, 2018, and April 24, 2023. Only inpatients (N = 57) admitted due to acute psychosis were included. All study participants provided written informed consent and were between 18 and 65 years old. Included patients had a primary diagnosis of schizophrenia, schizoaffective disorder, delusional disorder, or brief psychotic disorder, collectively referred to as SSD throughout the manuscript.

Exclusion criteria were as follows: diagnosis of psychotic disorders due to psychoactive substance use, concurrent clinically relevant neurological disorders, such as multiple sclerosis and epilepsy, history of encephalitis, meningitis, stroke, traumatic brain injury or cerebral surgery, current pregnancy or lactation, rheumatic disorders, inflammatory bowel disease, active malignancy, and acute or chronic infection.

### 2.2 Clinical assessments

The clinical characterization was performed by trained study personnel as previously described by our working group (Krčmář et al., 2023; Boudriot et al., 2024). The German version 7.0.2 of Mini International Neuropsychiatric Interview (M.I.N.I.) (Sheehan et al., 1998), based on DSM-5 criteria, was conducted with all study participants to confirm the diagnosis. Symptom severity was assessed with the Positive and Negative Syndrome Scale (PANSS) (Kay et al., 1987) and global functioning with the Global Assessment of Functioning (GAF) (Pedersen et al., 2018) scale. The assessments were performed within four weeks around the lumbar puncture. Information regarding medication, duration of illness (DUI), BMI, blood pressure, heart rate, concomitant somatic conditions, and current smoking status was collected based on self-report and by examining medical reports. Current treatment or history of clozapine use was used as a proxy for treatment resistance, as previously suggested (Pardiñas et al., 2022).

To assess the cognitive performance of the participants, the Montreal Cognitive Assessment (MoCA)(Nasreddine et al., 2005) and the Trail-Making-Test (TMT, part A and B) were performed in a subgroup of participants.

### 2.3 Blood and cerebrospinal fluid analyses

In line with recommendations from the German schizophrenia guideline (Campana et al., 2022; AWMF Leitlinienregister) lumbar puncture was offered to all patients with first- (FEP) or multi-episode psychosis (MEP), who had not yet received CSF analysis in the past as part of the diagnostic work-up to exclude concurrent somatic etiologies. Paired CSF and serum samples were analyzed as part of the clinical routine diagnostics by the Institute of Laboratory Medicine, LMU Munich.

Most of the study participants underwent a basic blood test, including full blood (N = 54) and serum (N = 52–56, depending on the variable assessed) analyses, within 3 weeks of the lumbar puncture as part of the clinical routine in our clinic. This was done during the morning hours under fasting conditions. The full blood analysis included a complete blood count, and the serum analysis included assessment of C-reactive protein (CRP), triglycerides, total cholesterol, low-density lipoprotein (LDL) cholesterol, high-density lipoprotein (HDL) cholesterol, glycated hemoglobin (HbA1c), albumin, immunoglobulin G (IgG) levels, and the presence of oligoclonal bands (OCBs). To compute CSF/serum albumin and IgG ratios, serum and CSF were collected and assessed at the same time point. The neutrophil-to-lymphocyte ratio (NLR) and monocyte-to-lymphocyte ratio (MLR) were calculated by dividing the absolute number of neutrophils and monocytes each by the absolute number of lymphocytes per individual (Steiner et al., 2020). Further information is provided in the supplementary methods section.

### 2.4 Magnetic resonance imaging

A subset of 28 participants underwent brain magnetic resonance imaging (MRI) using a Siemens Magnetom Prisma 3T scanner (Siemens Healthineers AG, Erlangen, Germany) equipped with a 32-channel head coil. Regional brain volumes, including the lateral ventricles, third and fourth ventricles, were quantified in cubic millimeters using FreeSurfer software (version 7.3.2; https://surfer.nmr.mgh.harvard.edu) (Fischl et al., 2002). We utilized the FreeSurfer atlas to obtain the ventricles' volumes. Additionally, the choroid plexus (ChP) in the lateral ventricles was manually segmented on the 3D-T1 images by one of the first authors (IJ), who was trained by a neuroimaging expert (DK). We employed ITK-SNAP software, version 4.2.0 (http://www.itksnap.org). The rater was blinded regarding clinical and imaging data and followed a previously published protocol for ChP segmentation (Bannai et al., 2023). The ChP as well as the ventricle measures were adjusted for total intracranial volume using the proportions method (Jernigan et al., 1982). Structural MRI data quality control was performed as previously described by our working group (Roell et al., 2024). Further information is provided in the supplementary methods section.

### 2.5 Assessment of BCB dysfunction

To quantify the integrity of the BCB, we computed a principal component analysis (PCA) including the CSF/serum albumin ratio, CSF/serum IgG ratio, and total protein levels in CSF as variables of interest (Fig. S1). All three measures have been associated with BCB disruption and regarded as biomarkers for BCB functioning (Rømer et al., 2023; Campana et al., 2024). Given their differing scales, the variables were scaled using the *“scale”* function in R, to increase comparability. PCA reduces the dimensionality of data that are correlated (Sanders-van Wijk et al., 2020). Instead of analysing all three measures individually, PCA summarizes the information represented by those measures in one BCB composite score, which mirrors the integrity of the BCB (the higher the BCB composite score, the lower the BCB integrity and the higher the degree of BCB dysfunction) better than any of those measures alone.

### 2.6 Statistical analyses

The R language (v4.2.1, R Core Team, 2021) in RStudio environment (RStudio Team, 2020) (Allaire)was used for all statistical analyses and visualizations.

To investigate the relationships between BCB composite score and measures of psychopathology, cognition, or cerebroventricular regions, we computed Bayesian linear and logistic regression models, using the *brms* package (Bürkner, 2017). Prior to conducting the Bayesian regression analyses, all continuous predictors and dependent variables were standardized (scaled to have a mean of zero and a standard deviation of one), using the *scale()* function in R. In the Bayesian linear regressions, BCB composite score, age, and sex were included as predictors, while the corresponding measures of psychopathology, cognition, and cerebroventricular regions served as dependent variables. In the Bayesian regression models, including cognition measures, we used years of education as an additional predictor. To investigate the association between BCB composite score and treatment resistance, we computed a Bayesian logistic regression model including BCB composite score, age, sex, smoking status, and BMI as predictors and history of clozapine treatment as a dependent variable. To study the relationships between cardiometabolic risk factors (e.g., total cholesterol, HbA1c) or inflammatory measures (e.g., CRP) and BCB composite score, we computed Bayesian linear regressions, including the respective cardiometabolic or inflammatory measure, along age, sex, BMI and smoker status (Asthana et al., 2010; Fernandes et al., 2016) as independent variables and the BCB composite score as a dependent variable.

Complementary frequentist analyses were conducted using linear and logistic regression models, with statistical inference based on a significance threshold of *p* < 0.05. Results from the descriptive statistics are shown as mean ± standard deviation (SD). Results from the Bayesian statistics are reported as scaled regression coefficients (β), along with their 95 % credible intervals (95 % CI) and Bayes factors (BF_10_). Details on the statistical analyses are reported in the Supplementary Information.

## 3 Results

### 3.1 Cohort characteristics

The study cohort consisted of 57 individuals with SSD who were inpatients at the Department of Psychiatry and Psychotherapy, LMU university hospital, Munich, and underwent a lumbar puncture for diagnostic reasons. The cohort included 42 (74 %) male and 15 (26 %) female participants with an average age of 34.32 ± 11.97 years. Nearly half of the participants (27/55; 49 %) were active smokers. Thirty-eight individuals were diagnosed with schizophrenia (67 %) and 13 with brief psychotic disorder (22 %), five with schizoaffective disorder (9 %), and one with delusional disorder (2 %). Thirty-five (61 %) of the participants had a first episode of psychosis at the time of inclusion. The mean duration of illness was 60.24 months (SD = 94.20), and the mean duration of antipsychotic treatment at the time of inclusion was 47.88 months (SD = 95.89). Forty-eight subjects (84.2 %) were treated with one or more antipsychotics, seventeen (29.8 %) with benzodiazepines, and eleven (19.3 %) with antidepressants (Table St3). None of the participants were treated with lipid-lowering agents (e.g., statins). Forty-seven participants were assessed with the PANSS and averaged a total score of 62.87 ± 13.09. The average GAF score of the participants was 46.87 ± 11.42. Forty-one individuals performed the cognitive tests scoring an average of 25.93 ± 3.65 in the MoCA. They required an average of 33.39 ± 14.83 s to complete the TMT-A and 94.38 ± 65.15 s for the TMT-B. One of the 41 participants did not complete the TMT-B (Table 1).

**Table 1:** Cohort characteristics.

	SSD	
**Demographic characteristics**	*Mean* ± *SD*	*N*
Age, years	34.32 ± 11.97	57
BMI	26.04 ± 4.09 57	
Education (years)	14.86 ± 4.10 41	
	*n* (%)	
Sex, male:female	42:15 (74 %) 57	
Current smoking, yes:no	27:28 (49 %)	55

**Clinical characteristics**	*Mean* ± *SD*	*N*
Duration of illness, months	60.24 ± 94.20	56
Duration of antipsychotic treatment, months	47.88 ± 95.89	56
PANSS positive symptoms	15.34 ± 4.14	47
PANSS negative symptoms	15.60 ± 5.75	47
PANSS general symptoms	31.83 ± 6.56	47
PANSS total score	62.87 ± 13.09	47
GAF	46.87 ± 11.42	47
TMT A time (seconds)	33.39 ± 14.83	41
TMT B time (seconds)	94.38 ± 65.15	40
MoCA score	25.93 ± 3.65	41
Systolic Blood Pressure (mmHg)	121.50 ± 10.55	57
	*n* (%)	
First episode psychosis, yes:no	35:22 (61.4 %)	57
Clozapine lifetime, yes:no	7:48 (12.7 %)	55
Diagnosis (*DSM-5*)	*n* (%)	
Schizophrenia	38 (66.7 %)	
Brief psychotic disorder	13 (22.8 %)	
Schizoaffective disorder	5 (8.8 %)	
Delusional disorder	1 (1.7 %)	

GAF, global assessment of functioning; MoCA, Montreal Cognitive Assessment; *N*, number of participants; PANSS, Positive and Negative Syndrome Scale; *SD*, standard deviation; SSD, Schizophrenia Spectrum Disorder; TMT, Trail making Test.

### 3.2 Cerebrospinal fluid and blood characteristics

The mean number of white blood cells was 0.88 ± 1.09/μl, and no participant demonstrated a CSF pleocytosis. Oligoclonal IgG bands were present in 5/57 (8.8 %) participants, and none of these cases were of intrathecal origin (Table St1). The mean CSF protein level was 42.42 ± 18.55 mg/dl, the mean CSF/serum albumin ratio (Q_alb_) was 6.53 ± 3.36, and the mean CSF/serum IgG ratio was 3.18 ± 1.66. The age-adjusted Q_alb_ was elevated in 24/57 (42.1 %) participants, and neuronal autoantibodies were not detected in CSF or blood in any of the participants.

Following our PCA, the first principal component (PC1) explained 98.5 % of the data variance (Fig. S1), so it was used to create a composite score that mirrors the BCB integrity (referred to as BCB composite score throughout the manuscript). Higher values of the BCB composite score indicate a higher level of disruption and lower BCB integrity.

In our cohort, the mean numbers of neutrophiles, monocytes, and lymphocytes (N = 56) were 4.27 ± 1.82 thou./μl, 0.53 ± 0.17 thou./μl, and 1.86 ± 0.57 thou./μl, respectively. The mean neutrophil-to-lymphocyte ratio (NLR) was 2.43 ± 1.03, the mean monocyte-to-lymphocyte ratio (MLR) was 0.30 ± 0.10, and the mean CRP was 0.18 ± 0.26 mg/dl. An elevated CRP level (>0.5 mg/dl) was found in 5/56 (8.9 %) participants. The mean total cholesterol was 178.20 ± 32.64 mg/dl, the mean high-density lipoprotein (HDL) cholesterol was 53.24 ± 16.48 mg/dl, the mean low-density lipoprotein (LDL) cholesterol was 109.10 ± 37.74 mg/dl. Those parameters were abnormal in 14/55 (25.5 %), 27/55 (49.1 %), and 19/55 (34.5 %) participants, respectively (Table St2). The mean triglyceride level was 118.50 ± 82.99 mg/dl, the mean glycated hemoglobin (HbA1c) was 5.34 ± 0.51 %. They were elevated in 14/55 (25.5 %) and 3/54 (5.6 %) participants, respectively.

### 3.3 Association between blood-cerebrospinal fluid barrier integrity and clinical phenotype

**Fig. 1:**
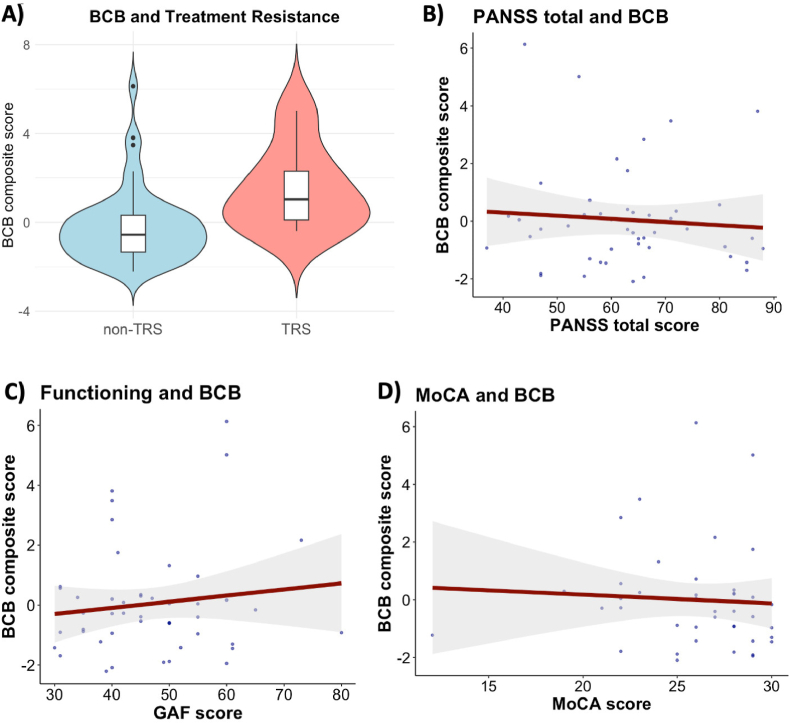
**Relationship between blood-cerebrospinal fluid barrier integrity and clinical phenotype.** **(A)** Comparison of mean blood-cerebrospinal fluid barrier composite score between treatment-resistant schizophrenia group (red) and non-treatment-resistant schizophrenia group (turquoise) illustrated with box and violin plots. Groups were compared using a Bayesian logistic regression model, controlling for age, sex, and BMI. N_non-TRS_ = 48, N_TRS_ = 7. Regression plots illustrating associations between blood-cerebrospinal fluid barrier composite score, **(A)** PANSS total score (N = 47), **(B)** GAF score (N = 47) and **(C)** MoCA score (N = 35). Multiple Bayesian linear regressions were employed, controlling for covariates. Abbreviations: N, number of participants; BCB, blood-cerebrospinal fluid barrier; TRS, treatment-resistant schizophrenia; non-TRS, non-treatment-resistant schizophrenia; PANSS, Positive and Negative Syndrome Scale; GAF, Global Assessment of Functioning scale; MoCA, Montreal Cognitive Assessment scale. (For interpretation of the references to colour in this figure legend, the reader is referred to the Web version of this article.)

First, we investigated whether there was an association between psychopathology or level of functioning and BCB integrity. We found no evidence of associations between BCB composite score and PANSS positive, negative, general, and total scores or GAF score (Fig. 1B and C, Fig. S2, Table S2). However, there was moderate evidence for an association between current or prior treatment with clozapine and higher BCB composite score (Fig. 1A) (estimate [95 % CI] = 1.18 [−0.005, 2.342]; BF_10_ = 4.127). We found no evidence of associations between TMT A, TMT B, MoCA scores, and BCB composite score (Fig. 1D, Fig. S2, Table S3). Furthermore, we found no evidence of associations between BCB composite score and duration of illness, duration of antipsychotic treatment, or first episode psychosis status (Fig. S3, Table S1). Sensitivity analyses in a subgroup of individuals with schizophrenia and schizoaffective disorders provided similar results (Table S8–S10).

### 3.4 Relationship between cardiometabolic factors and blood-cerebrospinal fluid barrier integrity

**Fig. 2:**
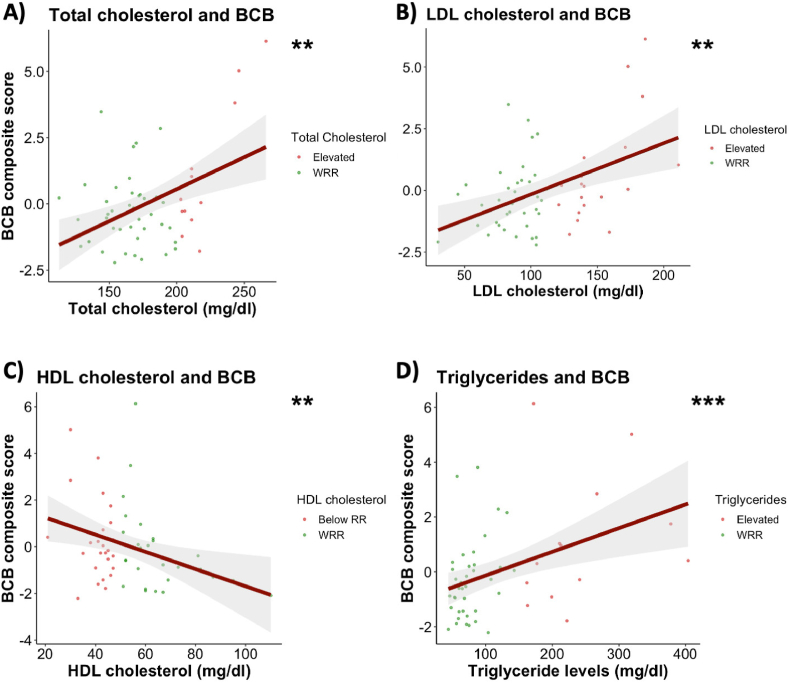
**Association between blood-cerebrospinal fluid barrier integrity and serum lipids.** Regression plots illustrating associations between blood-cerebrospinal fluid barrier composite score, **(A)** total cholesterol, **(B)** LDL cholesterol, **(C)** HDL cholesterol and **(D)** triglycerides. Multiple Bayesian linear regressions were employed, controlling for age, sex, smoking status, and BMI. N = 53. Abbreviations: N, number of participants; BCB, blood-cerebrospinal fluid barrier; LDL cholesterol, low-density lipoprotein cholesterol; HDL cholesterol, high-density lipoprotein cholesterol; WRR, within reference range.

Subsequently, we explored the relationships between cardiometabolic risk factors and the blood-cerebrospinal fluid barrier composite score in SSD participants. We found extreme evidence of positive associations between total cholesterol (estimate [95 % CI] = 0.807 [0.350, 1.262]; BF_10_ = 126.1), LDL cholesterol (estimate [95 % CI] = 0.812 [0.376, 1.25]; BF_10_ = 1084), triglycerides (estimate [95 % CI] = 0.992 [0.577, 1.406]; BF_10_ = 10 103) on the one hand, and BCB composite score on the other. Moreover, there was very strong evidence of a negative association between HDL cholesterol (estimate [95 % CI] = −0.703 [−1.132, −0.269]; BF_10_ = 33.3) and BCB composite score (Fig. 2, Table S4). There was no evidence of associations between other cardiovascular factors, such as systolic blood pressure or HbA1c, and BCB composite score (Fig. S4, Table S4). Sensitivity analyses in a subgroup of individuals with schizophrenia and schizoaffective disorders provided similar results (Table S11).

### 3.5 Relationship between peripheral inflammation and blood-cerebrospinal fluid barrier integrity

**Fig. 3:**
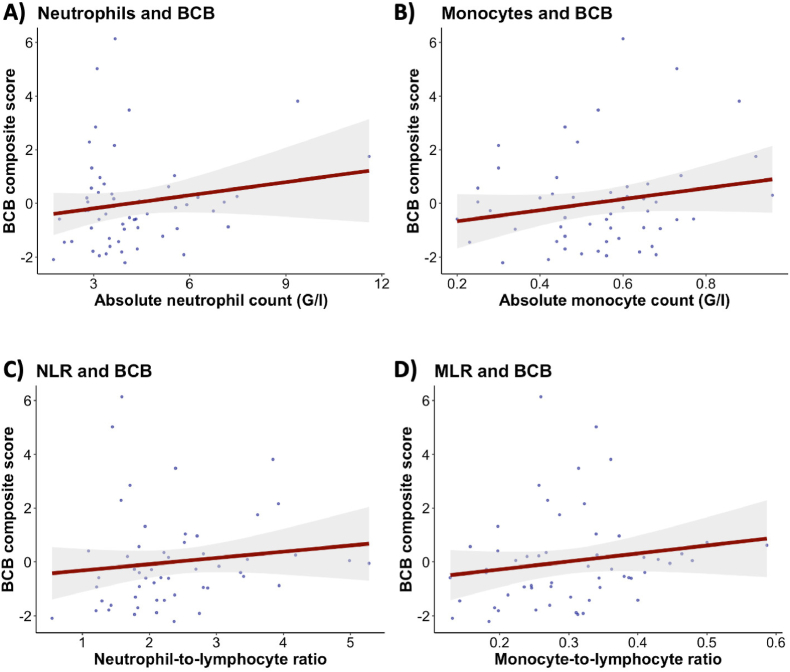
Relationship between blood-cerebrospinal fluid barrier integrity and peripheral inflammatory measures. Regression plots illustrating associations between blood-cerebrospinal fluid barrier composite score, **(A)** neutrophil count, **(B)** monocyte count, **(C)** neutrophil-to-lymphocyte ratio and **(D)** monocyte-to-lymphocyte ratio. Multiple Bayesian linear regressions were employed, controlling for age, sex, smoking status, and BMI. N = 54. Abbreviations: N, number of participants; BCB, blood-cerebrospinal fluid barrier; NLR, neutrophil-to-lymphocyte ratio; MLR, monocyte-to-lymphocyte ratio.

Next, we investigated a possible association between peripheral inflammatory factors and BCB integrity. We did not find evidence of a relationship between absolute neutrophil, monocyte, and lymphocyte counts and the BCB composite score, respectively. There was also no evidence of associations between NLR, MLR, CRP levels, and BCB composite score (Fig. 3, Fig. S5, Table S5). Sensitivity analyses in a subgroup of individuals with schizophrenia and schizoaffective disorders provided similar results (Table S12).

### 3.6 Relationship between blood-cerebrospinal fluid barrier integrity and cerebroventricular regions

**Fig. 4:**
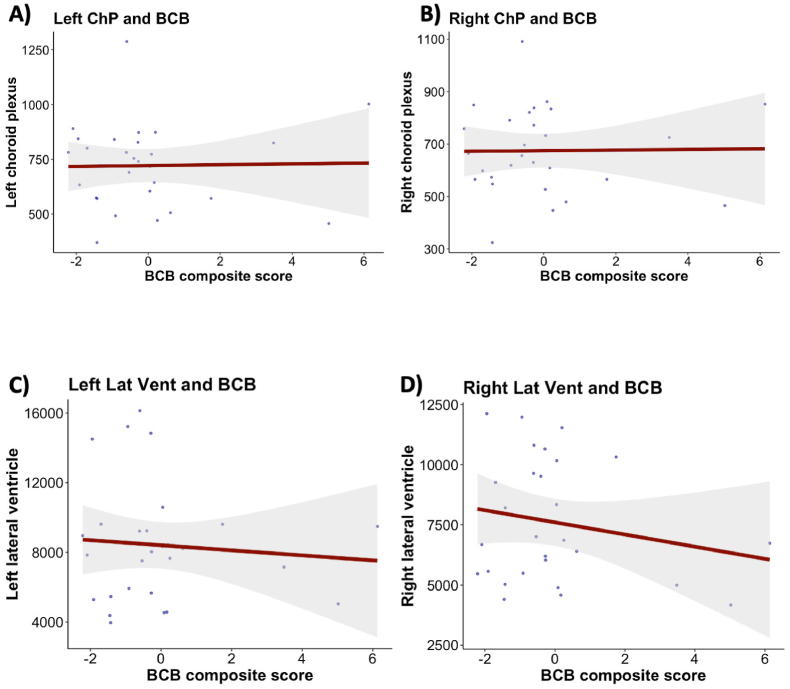
Relationship between blood-cerebrospinal fluid barrier integrity and cerebroventricular regions. Regression plots illustrating associations between blood-cerebrospinal fluid barrier composite score, **(A)** left choroid plexus volume, **(B)** right choroid plexus volume, **(C)** left lateral ventricle volume, and **(D)** right lateral ventricle volume. Multiple Bayesian linear regressions were employed, controlling for age and sex. N = 28. Abbreviations: N, number of participants; BCB, blood-cerebrospinal fluid barrier; Left ChP, left choroid plexus; Right ChP, right choroid plexus; Left Lat Vent, left lateral ventricle; Right Lat Vent, right lateral ventricle.

Due to the functional relationship between the BCB and the cerebroventricular regions, particularly the choroid plexus, we investigated the association between the level of BCB disruption and the volumes of the lateral ventricles, the 3rd ventricle, the 4th ventricle, and the choroid plexus in a subset of participants. We found no evidence of associations between the BCB composite score and the volumes of the 3rd ventricle, the 4th ventricle, the left and right lateral ventricle, or the left and right choroid plexus (Fig. 4, S6, Table S6). Sensitivity analyses in a statistical model, including 10.13039/501100013934BMI as an additional covariate (Table S7), and in a subgroup of individuals with schizophrenia and schizoaffective disorders (Table S13), supported our initial findings.

## 4 Discussion

In our study, we found extreme evidence of associations between higher total cholesterol, LDL cholesterol, triglycerides, and disrupted BCB integrity, as well as very strong evidence of an association between lower HDL cholesterol and disrupted BCB integrity in individuals with SSD. We found moderate evidence of a higher degree of BCB disruption in individuals with pharmacological treatment resistance, but did not find evidence of associations between BCB composite score and other measures of psychopathology or disease characteristics. Additionally, there was no evidence of a relationship between BCB disruption and peripheral immune markers or volumetric measures of cerebroventricular regions, respectively.

BCB disruption is a common finding in SSD (Campana et al., 2022), but data on its relevance regarding psychopathology and disease course remains scarce. Even though some previous studies have investigated the link between abnormal CSF/serum albumin ratio (a proxy for BCB impairment) and different measures of symptomatology, such as cognitive deficits (Maurus et al., 2023), positive, negative, or general symptoms, no significant associations could be found across both sexes. Interestingly, Oviedo-Salcedo, Wagner et al. found a trend towards associations between elevated CSF protein, CSF/serum albumin ratio, and history of treatment with clozapine (a proxy for TRS), that did not reach statistical significance (Oviedo-Salcedo et al., 2021). Despite the weak evidence, it aligns with our results, indicating a higher degree of BCB disruption in individuals with a history of clozapine treatment. This data must be regarded as preliminary and interpreted cautiously (not only due to a Bayesian factor suggesting moderate evidence, but also due to the low number of individuals with a history of clozapine treatment (N = 7). A recent individual participant data meta-analysis from our working group (Campana et al., 2024) demonstrated that male individuals with SSD and elevated CSF/serum albumin ratio have significantly higher PANSS positive scores than male individuals with SSD and CSF/serum albumin ratio within the reference range. Of note, clozapine is usually prescribed to patients with treatment-resistant positive symptoms, which aligns well with these data. Overall, the results from our study and others suggest that BCB impairment might be associated with a higher degree of symptom severity and even treatment resistance, but should be interpreted carefully in light of the discussed limitations.

Dyslipidemia occurs in a substantial proportion of individuals with SSD (Mitchell et al., 2013). To the best of our knowledge, this is the first study to show evidence of a relationship between blood lipid levels and BCB integrity in SSD. In line with our findings, a previous study found that subjects with Alzheimer's disease and elevated CSF/serum albumin ratio (a proxy for BCB impairment) had significantly higher mean plasma triglycerides and lower mean HDL cholesterol than individuals without BCB impairment (Bowman et al., 2012). Also, higher levels of serum HDL cholesterol were associated with a lower prevalence of BCB disruption in multiple sclerosis patients (Fellows et al., 2015). Data from healthy participants is very scarce. A study including healthy middle-aged individuals found that high BMI and waist-hip ratio predicted BCB impairment 20 years later. Interestingly, they didn't find an association between hyperlipidemia and BCB impairment (Janelidze et al., 2017). Taken together, the data from our and previous studies suggest that dyslipidemia in neuropsychiatric conditions such as SSD or Alzheimer's disease might affect BCB functioning differently than in healthy populations. Nevertheless, since age impacts the BCB (Castellazzi et al., 2024), the generalizability of these findings, especially in younger individuals, remains limited. Preclinical evidence also points to interactions between CNS barriers and peripheral lipids (Rhea and Banks, 2021). For example, LDLr-knock-out mice, fed a high cholesterol diet, were more susceptible to blood-brain barrier damage and cognitive deficits (de Oliveira et al., 2020). In two other studies, dietary-fat-induced blood-brain barrier dysfunction was restored via treatment with statins (Jiang et al., 2012; Pallebage-Gamarallage et al., 2012) or ibuprofen (Pallebage-Gamarallage et al., 2012). It has been suggested that lipids affect the structure and permeability of CNS barriers by altering the brain endothelial cells (Rhea and Banks, 2021). Indeed, the effects of dyslipidemia on blood vessels and, particularly, its contribution to atherosclerosis are well-studied (Libby et al., 2019). Although we could not investigate the directionality or possible mechanisms of the association between blood lipid levels and BCB disruption, it is possible that dyslipidemia in individuals with SSD damages the endothelial cells of the vessels, forming the BCB, thus affecting its integrity. A previous study investigating the impact of hypercholesterolemia on choroid plexus epithelial cells in rabbits demonstrated that cholesterol insults from the circulation induce dysfunction of choroid plexus epithelial cells (Obata and Narita, 2020). This is particularly interesting since the choroid plexus epithelial cells are an integral building block of the BCB (Yakimov et al., 2023). Furthermore, some evidence suggests that triglycerides are found in low amounts in the human CSF and can cross the BBB and BCB in mice (Banks et al., 2018).

Previous studies in rats and humans have suggested that hypertension can disrupt the BCB or BBB integrity (González-Marrero et al., 2013; van den Kerkhof et al.) and hyperglycemia can exacerbate BBB disruption (Banks, 2020), potentially through inflammatory pathways (Wei et al., 2023). In our study, we did not find evidence of associations between systolic blood pressure, glycated hemoglobin, and BCB composite score, respectively. However, even though the prevalence of cardiovascular diseases is increased in individuals with SSD, most of our participants did not have overt diabetes or hypertension. Thus, follow-up studies in cohorts enriched for cardiovascular comorbidities are needed. Furthermore, we only included systolic blood pressure from a single measurement, which could vary substantially and be influenced by multiple factors.

Building on evidence from multiple sclerosis (Hansen et al., 2024) and Alzheimer's research (Liebner et al., 2018), as well as on the inflammatory hypothesis for schizophrenia (Owen et al., 2016), some authors have suggested that CNS barrier disruption arises as a consequence of inflammatory insult and/or subtle immune dysregulation (Pollak et al., 2018). Even though abnormal inflammatory markers are evident in individuals with SSD, both in plasma (Halstead et al., 2023) and CSF (Rømer et al., 2023), a previous study trying to link peripheral inflammation (CRP) to BCB impairment failed (Campana et al., 2022). In our current study, we also did not find evidence of associations between peripheral leukocytes, CRP, and BCB integrity, confirming previous evidence. It is possible that markers of inflammation (non-high-sensitive CRP and immune cell counts) used in both studies were not sensitive enough to detect subtle immune dysregulation and its link to BCB impairment. To overcome this limitation, future studies need to conduct a deeper immunoprofiling of blood and CSF, including inflammatory proteins such as cytokines.

Anatomically, the BCB is formed by epithelial cells of the choroid plexus, fenestrated blood vessels, and the arachnoid membrane facing the CSF (Tumani et al., 2017). Interestingly, emerging evidence points to morphological alterations of the choroid plexus in psychosis (Lizano et al., 2019), and ventricular enlargement is a well-known phenomenon in some individuals with SSD (Jauhar et al., 2022). Thus, these cerebroventricular regions and BCB integrity seem to be affected in individuals with SSD; however, to the best of our knowledge, the relationship between these variables has never been investigated before in any mental disorder. In a recent study of individuals with amyotrophic lateral sclerosis, the authors showed higher choroid plexus volumes compared to healthy controls and found a significant positive correlation between choroid plexus volume and CSF/serum albumin ratio (as a proxy for BCB disruption) (Dai et al., 2024). In our study of people with SSD, we found no evidence of associations between BCB integrity and the choroid plexus or any of the cerebral ventricles.

The limitations of our study include its cross-sectional design, which does not allow us to study disease progression or treatment response/remission in relation to BCB integrity or its potential temporal dynamics. Furthermore, a relevant part of the study participants was treated with antipsychotics, potentially influencing plasma lipid levels (Meyer and Koro, 2004) and BCB integrity (Elmorsy et al., 2014). It is not clear whether treatment resistance per se or clozapine-induced adverse reactions drive the observed association between history of clozapine treatment and BCB dysfunction, since treatment with clozapine is strongly associated with dyslipidemia (Henderson, 2001). Another important limitation is that blood for immunological and cardiometabolic analyses was taken within a three-week period around the lumbar puncture. Consequently, we might have missed associations between increased BCB permeability and peripheral measures that are not stable over time, such as immune cell counts or CRP. Even though this is the first study to investigate such an association in people with SSD, the sample size was relatively small (N = 28), and subsequent well-powered studies need to address this question.

Our study adds to the growing body of literature pointing to the relevance of brain-body interactions and CNS barrier impairment for the pathophysiology of SSD. Consequently, addressing cardiometabolic factors in individuals with SSD might have implications that extend beyond physical health and influence the brain as well as the course of the disease. Future investigations with sufficiently powered cohorts, deeper immunometabolic phenotyping performed in both blood and CSF, and longitudinal designs might help elucidate the aetiology and clinical relevance of BCB disruption in individuals with SSD.

## CRediT authorship contribution statement

**Vladislav Yakimov:** Writing – review & editing, Writing – original draft, Visualization, Validation, Supervision, Project administration, Methodology, Investigation, Formal analysis, Data curation, Conceptualization. **Iris Jäger:** Writing – review & editing, Writing – original draft, Visualization, Validation, Investigation, Formal analysis, Data curation, Conceptualization. **Lukas Roell:** Writing – review & editing, Validation, Supervision, Methodology, Investigation. **Emanuel Boudriot:** Writing – review & editing, Supervision, Methodology, Investigation, Data curation. **Verena Meisinger:** Writing – review & editing, Investigation, Data curation. **Mattia Campana:** Writing – review & editing, Supervision, Data curation. **Lenka Krčmář:** Writing – review & editing, Supervision, Data curation. **Sean Halstead:** Writing – review & editing, Methodology. **Nicola Warren:** Writing – review & editing, Methodology. **Dan Siskind:** Writing – review & editing, Methodology. **Isabel Maurus:** Writing – review & editing, Supervision. **Alkomiet Hasan:** Writing – review & editing, Supervision, Resources, Funding acquisition. **Peter Falkai:** Writing – review & editing, Supervision, Resources, Funding acquisition. **Andrea Schmitt:** Writing – review & editing, Supervision, Resources, Funding acquisition. **Florian J. Raabe:** Writing – review & editing, Supervision, Resources, Funding acquisition. **Daniel Keeser:** Writing – review & editing, Supervision, Resources, Methodology, Funding acquisition. **Elias Wagner:** Writing – review & editing, Writing – original draft, Supervision, Resources, Methodology, Investigation, Funding acquisition, Data curation, Conceptualization. **Joanna Moussiopoulou:** Writing – review & editing, Writing – original draft, Supervision, Resources, Methodology, Investigation, Funding acquisition, Data curation.

## Funding

The study was endorsed by the 10.13039/501100002347Federal Ministry of Education and Research (Bundesministerium für Bildung und Forschung [10.13039/501100002347BMBF]) within the initial phase of the German Center for Mental 10.13039/100018696Health (DZPG) (grant: 01 EE2303C to 10.13039/501100020211AH, and 01EE2303A, 01EE2303F to PF, AS). This research was supported by 10.13039/501100002347BMBF with the EraNet project GDNF UpReg (01EW2206) to VY and PF. VY was supported by the Residency/PhD track of the 10.13039/100018173International Max Planck Research School for Translational Psychiatry (IMPRS-10.13039/501100017484TP) and by the Faculty of Medicine at 10.13039/501100005722LMU Munich (FöFoLe Reg.-Nr. 1226/2024). JM was supported by the Faculty of Medicine at 10.13039/501100005722LMU Munich (FöFoLe Reg.-Nr. 1167). The study was supported by the EU HORIZON-INFRA-2024-TECH-01-04 project DTRIP4H 101188432 to PF, AS, and FR. The procurement of the Prisma 3T MRI scanner was supported by the 10.13039/501100001659Deutsche Forschungsgemeinschaft (10.13039/501100001659DFG, INST 86/1739-1 FUGG). No funding was received by commercial or not-for-profit sectors. IJ and VM were supported by doctoral scholarships from the Faculty of Medicine, 10.13039/501100005722LMU Munich, Munich, Germany. LK, and IM were supported by the Else Kröner-Fresenius Foundation for the Residency/PhD track of the 10.13039/100018173International Max Planck Research School for Translational Psychiatry (IMPRS-10.13039/501100017484TP), Munich, Germany.

## Declaration of competing interest

During the preparation of this work the author(s) used the GPT – 4 model developed by OpenAI in order to improve readability and language of the manuscript. After using this tool, the author(s) reviewed and edited the content as needed and take(s) full responsibility for the content of the publication. The authors declare that they have no biomedical financial interests or potential conflicts of interest regarding the content of this report. AH received paid speakership by Janssen, Otsuka, Lundbeck, and Recordati and was member of advisory boards of these companies and Rovi. PF received paid speakership by Boehringer-Ingelheim, Janssen, Otsuka, Lundbeck, Recordati, and Richter and was member of advisory boards of these companies and Rovi. EW was invited to advisory boards from Recordati, Teva and Boehringer-Ingelheim. 10.13039/100024172SH is supported by an Australian Research Training Program scholarship. D.S. is supported by an 10.13039/501100000925NHMRC Investigator Fellowship GNT 1194635. 10.13039/100018068NW has received speaker fees from 10.13039/100019120Otsuka, 10.13039/501100013327Lundbeck and Janssen. All other authors report no biomedical financial interests or potential conflicts of interest.

## Data Availability

The documentation sheets including the de-identified data will be made available upon publication on OSF (https://osf.io/27cra/). All software packages used in this study are publicly available.

## References

[bib1] Alberti K.G.M.M., Eckel R.H., Grundy S.M. (2009). Harmonizing the metabolic syndrome: a joint interim statement of the international diabetes federation task force on epidemiology and prevention; national heart, lung, and blood Institute; American heart association; world heart federation; international atherosclerosis society; and international association for the study of obesity. Circulation (New York, N. Y.).

[bib2] Allaire JJ. RStudio: Integrated Development Environment for R.

[bib3] Asthana A., Johnson H.M., Piper M.E., Fiore M.C., Baker T.B., Stein J.H. (2010). Effects of smoking intensity and cessation on inflammatory markers in a large cohort of active smokers. Am. Heart J..

[bib4] AWMF leitlinienregister. https://register.awmf.org/de/leitlinien/detail/038-009.

[bib5] Banks W.A. (2020). The blood-brain barrier interface in diabetes mellitus: dysfunctions, mechanisms and approaches to treatment. Curr. Pharm. Des..

[bib6] Banks W.A., Farr S.A., Salameh T.S. (2018). Triglycerides cross the blood–brain barrier and induce central leptin and insulin receptor resistance. Int. J. Obes..

[bib7] Bannai D., Cao Y., Keshavan M., Reuter M., Lizano P. (2023). Manual segmentation of the human choroid plexus using brain MRI. J. Vis. Exp..

[bib8] Benros M.E., Nielsen P.R., Nordentoft M., Eaton W.W., Dalton S.O., Mortensen P.B. (2011). Autoimmune diseases and severe infections as risk factors for schizophrenia: a 30-year population-based register study. Am. J. Psychiatr..

[bib9] Benros M.E., Eaton W.W., Mortensen P.B. (2014). The epidemiologic evidence linking autoimmune diseases and psychosis. Biol. Psychiatry.

[bib10] Bioque M., García-Portilla M. a P., García-Rizo C. (2018). Evolution of metabolic risk factors over a two-year period in a cohort of first episodes of psychosis. Schizophr. Res..

[bib11] Bishop J.R., Zhang L., Lizano P. (2022). Inflammation subtypes and translating inflammation-related genetic findings in schizophrenia and related psychoses: a perspective on pathways for treatment stratification and novel therapies. Harv. Rev. Psychiatr..

[bib12] Bora E., Akdede B.B., Alptekin K. (2017). The relationship between cognitive impairment in schizophrenia and metabolic syndrome: a systematic review and meta-analysis. Psychol. Med..

[bib13] Boudriot E, Gabriel V, Popovic D, et al. Signature of altered retinal microstructures and electrophysiology in schizophrenia spectrum disorders is associated with disease severity and polygenic risk. Biol. Psychiatry. Published online April 26, 2024:S0006-3223(24)01262-9. doi:10.1016/j.biopsych.2024.04.014. 10.1016/j.biopsych.2024.04.014 38679358

[bib14] Bowman G.L., Kaye J.A., Quinn J.F. (2012). Dyslipidemia and blood-brain barrier integrity in Alzheimer's disease. Current Gerontology and Geriatrics Research.

[bib15] Bürkner P.C. (2017). Brms: an R package for bayesian multilevel models using stan. J. Stat. Software.

[bib16] Campana M., Strauß J., Münz S. (2022). Cerebrospinal fluid pathologies in schizophrenia-spectrum disorder—a retrospective chart review. Schizophr. Bull..

[bib17] Campana M., Yakimov V., Moussiopoulou J. (2024). Association of symptom severity and cerebrospinal fluid alterations in recent onset psychosis in schizophrenia-spectrum disorders - an individual patient data meta-analysis. Brain Behav. Immun..

[bib18] Castellazzi M., Candeloro R., Trevisan C. (2024). Sex differences in albumin quotient and cerebrospinal fluid total protein content do not depend on anthropometric factors. J. Personalized Med..

[bib19] Dai T., Lou J., Kong D. (2024). Choroid plexus enlargement in amyotrophic lateral sclerosis patients and its correlation with clinical disability and blood-CSF barrier permeability. Fluids Barriers CNS.

[bib20] de Oliveira J., Engel D.F., de Paula G.C. (2020). High cholesterol diet exacerbates blood-brain barrier disruption in LDLr–/– mice: impact on cognitive function. J. Alzheim. Dis..

[bib21] Elmorsy E., Elzalabany L.M., Elsheikha H.M., Smith P.A. (2014). Adverse effects of antipsychotics on micro-vascular endothelial cells of the human blood–brain barrier. Brain Res..

[bib22] Fellows K., Uher T., Browne R.W. (2015). Protective associations of HDL with blood-brain barrier injury in multiple sclerosis patients. JLR (J. Lipid Res.).

[bib23] Fernandes B.S., Steiner J., Bernstein H.G. (2016). C-reactive protein is increased in schizophrenia but is not altered by antipsychotics: meta-analysis and implications. Mol. Psychiatr..

[bib24] Fischl B., Salat D.H., Busa E. (2002). Whole brain segmentation: automated labeling of neuroanatomical structures in the human brain. Neuron.

[bib25] González-Marrero I., Castañeyra-Ruiz L., González-Toledo J.M. (2013). High blood pressure effects on the blood to cerebrospinal fluid barrier and cerebrospinal fluid protein composition: a two-dimensional electrophoresis study in spontaneously hypertensive rats. Int. J. Hypertens..

[bib26] Hagi K., Nosaka T., Dickinson D. (2021). Association between cardiovascular risk factors and cognitive impairment in people with schizophrenia: a systematic review and meta-analysis. JAMA Psychiatry.

[bib27] Halstead S., Siskind D., Amft M. (2023). Alteration patterns of peripheral concentrations of cytokines and associated inflammatory proteins in acute and chronic stages of schizophrenia: a systematic review and network meta-analysis. Lancet Psychiatry.

[bib28] Hansen C.E., Kamermans A., Mol K. (2024). Inflammation-induced TRPV4 channels exacerbate blood-brain barrier dysfunction in multiple sclerosis. J. Neuroinflammation.

[bib29] Henderson D.C. (2001). Clozapine: diabetes mellitus, weight gain, and lipid abnormalities. J. Clin. Psychiatry.

[bib30] Hjorthøj C., Stürup A.E., McGrath J.J., Nordentoft M. (2017). Years of potential life lost and life expectancy in schizophrenia: a systematic review and meta-analysis. Lancet Psychiatry.

[bib31] Janelidze S., Hertze J., Nägga K. (2017). Increased blood-brain barrier permeability is associated with dementia and diabetes but not amyloid pathology or APOE genotype. Neurobiol. Aging.

[bib32] Jauhar S., Johnstone M., McKenna P.J. (2022). Schizophrenia. Lancet..

[bib33] Jernigan T.L., Zatz L.M., Moses J.A., Berger P.A. (1982). Computed tomography in schizophrenics and normal volunteers. I. Fluid volume. Arch. Gen. Psychiatry.

[bib34] Jiang X., Guo M., Su J. (2012). Simvastatin blocks blood-brain barrier disruptions induced by elevated cholesterol both in vivo and in vitro. Int. J. Alzheimers Dis..

[bib36] Kalman J.L., Burkhardt G., Adorjan K. (2022). Biobanking in everyday clinical practice in psychiatry-The Munich Mental Health Biobank. Front. Psychiatr..

[bib37] Kay S.R., Fiszbein A., Opler L.A. (1987). The positive and negative syndrome scale (PANSS) for schizophrenia. Schizophr. Bull..

[bib38] Kim S., Hwang Y., Lee D., Webster M.J. (2016). Transcriptome sequencing of the choroid plexus in schizophrenia. Transl. Psychiatry.

[bib39] Krčmář L., Jäger I., Boudriot E. (2023). The multimodal Munich Clinical Deep Phenotyping study to bridge the translational gap in severe mental illness treatment research. Front. Psychiatr..

[bib40] Libby P., Buring J.E., Badimon L. (2019). Atherosclerosis. Nat. Rev. Dis. Primers.

[bib41] Liebner S., Dijkhuizen R.M., Reiss Y., Plate K.H., Agalliu D., Constantin G. (2018). Functional morphology of the blood-brain barrier in health and disease. Acta Neuropathol..

[bib42] Lizano P., Lutz O., Ling G. (2019). Association of choroid plexus enlargement with cognitive, inflammatory, and structural phenotypes across the psychosis spectrum. Aust. J. Pharm..

[bib43] Maurus I., Wagner S., Campana M. (2023). The relationship between blood–brain barrier dysfunction and neurocognitive impairments in first-episode psychosis: findings from a retrospective chart analysis. BJPsych Open.

[bib44] McWhinney S.R., Brosch K., Calhoun V.D. (2022). Obesity and brain structure in schizophrenia – ENIGMA study in 3021 individuals. Mol. Psychiatr..

[bib45] Meyer J.M., Koro C.E. (2004). The effects of antipsychotic therapy on serum lipids: a comprehensive review. Schizophr. Res..

[bib46] Mitchell A.J., Vancampfort D., Sweers K., van Winkel R., Yu W., De Hert M. (2013). Prevalence of metabolic syndrome and metabolic abnormalities in schizophrenia and related disorders--a systematic review and meta-analysis. Schizophr. Bull..

[bib47] Moussiopoulou J, Yakimov V, Roell L, et al. Higher blood-brain barrier leakage in schizophrenia-spectrum disorders: a comparative dynamic contrast-enhanced magnetic resonance imaging study with healthy controls. Brain Behav. Immun. Published online April 5, 2025:S0889-1591(25)00125-4. doi:10.1016/j.bbi.2025.04.003. 10.1016/j.bbi.2025.04.003 40194748

[bib48] Nasreddine Z.S., Phillips N.A., Bédirian V. (2005). The Montreal Cognitive Assessment, MoCA: a brief screening tool for mild cognitive impairment. J. Am. Geriatr. Soc..

[bib49] Obata F., Narita K. (2020). Hypercholesterolemia negatively influences morphology and molecular markers of epithelial cells within the choroid plexus in rabbits. Fluids Barriers CNS.

[bib50] Obermeier B., Daneman R., Ransohoff R.M. (2013). Development, maintenance and disruption of the blood-brain barrier. Nat. Med..

[bib51] Oviedo-Salcedo T., Wagner E., Campana M. (2021). Cerebrospinal fluid abnormalities in first- and multi-episode schizophrenia-spectrum disorders: impact of clinical and demographical variables. Transl. Psychiatry.

[bib52] Owen M.J., Sawa A., Mortensen P.B. (2016). Schizophrenia. Lancet.

[bib53] Pallebage-Gamarallage M., Lam V., Takechi R., Galloway S., Clark K., Mamo J. (2012). Restoration of dietary-fat induced blood-brain barrier dysfunction by anti-inflammatory lipid-modulating agents. Lipids Health Dis..

[bib54] Pardiñas A.F., Smart S.E., Willcocks I.R. (2022). Interaction testing and polygenic risk scoring to estimate the association of common genetic variants with treatment resistance in schizophrenia. JAMA Psychiatry.

[bib55] Pedersen G., Urnes Ø., Hummelen B., Wilberg T., Kvarstein E.H. (2018). Revised manual for the global assessment of functioning scale. Eur. Psychiatry.

[bib56] Pollak T.A., Drndarski S., Stone J.M., David A.S., McGuire P., Abbott N.J. (2018). The blood-brain barrier in psychosis. Lancet Psychiatry.

[bib57] Rhea E.M., Banks W.A. (2021). Interactions of lipids, lipoproteins, and apolipoproteins with the blood-brain barrier. Pharm. Res..

[bib58] Rødevand L., Rahman Z., Hindley G.F.L. (2023). Characterizing the shared genetic underpinnings of schizophrenia and cardiovascular disease risk factors. Aust. J. Pharm..

[bib59] Roell L., Keeser D., Papazov B. (2024). Effects of exercise on structural and functional brain patterns in schizophrenia-data from a multicenter randomized-controlled study. Schizophr. Bull..

[bib60] Rømer T.B., Jeppesen R., Christensen R.H.B., Benros M.E. (2023). Biomarkers in the cerebrospinal fluid of patients with psychotic disorders compared to healthy controls: a systematic review and meta-analysis. Mol. Psychiatr..

[bib61] Sanders-van Wijk S., Tromp J., Beussink-Nelson L. (2020). Proteomic evaluation of the comorbidity-inflammation paradigm in heart failure with preserved ejection fraction. Circulation (New York, N. Y.).

[bib62] Sayed S.E., Gomaa S., Alhazmi A., ElKalla I., Khalil D. (2023). Metabolic profile in first episode drug naïve patients with psychosis and its relation to cognitive functions and social cognition: a case control study. Sci. Rep..

[bib63] Sheehan D.V., Lecrubier Y., Sheehan K.H. (1998). The Mini-International Neuropsychiatric Interview (M.I.N.I.): the development and validation of a structured diagnostic psychiatric interview for DSM-IV and ICD-10. J. Clin. Psychiatry.

[bib64] Steiner J., Frodl T., Schiltz K. (2020). Innate immune cells and C-reactive protein in acute first-episode psychosis and schizophrenia: relationship to psychopathology and treatment. Schizophr. Bull..

[bib65] (2018). The blood–brain barrier in psychosis. Lancet Psychiatry.

[bib66] Trubetskoy V., Pardiñas A.F., Qi T. (2022). Mapping genomic loci implicates genes and synaptic biology in schizophrenia. Nature.

[bib67] Tumani H., Huss A., Bachhuber F. (2017). The cerebrospinal fluid and barriers - anatomic and physiologic considerations. Handb. Clin. Neurol..

[bib69] Wei X., Xing Z., Huang T., Zhang M., Song J., Zhao Y. (2023). Hyperglycemia disrupted the integrity of the blood-brain barrier following diffuse axonal injury through the sEH/NF-κB pathway. Immun. Inflamm. Dis..

[bib70] Williams M.R., Macdonald C.M., Turkheimer F.E. (2023). Histological examination of choroid plexus epithelia changes in schizophrenia. Brain Behav. Immun..

[bib71] Xiao P., Li C., Mi J., Wu J. (2024). Evaluating the distinct effects of body mass index at childhood and adulthood on adult major psychiatric disorders. Sci. Adv..

[bib72] Yakimov V., Moussiopoulou J., Hasan A., Wagner E. (2023). The common misconception of blood–brain barrier terminology in psychiatry and neurology. Eur. Arch. Psychiatr. Clin. Neurosci..

[bib73] Yakimov V., Moussiopoulou J., Roell L. (2024). Investigation of choroid plexus variability in schizophrenia-spectrum disorders-insights from a multimodal study. Schizophrenia (Heidelb)..

