# Supplementary material for: Relationship between blood-cerebrospinal fluid barrier integrity, cardiometabolic, and inflammatory factors in schizophrenia-spectrum disorders
Source: Brain Behav Immun Health. 2025 May 27;47:101024. doi: 10.1016/j.bbih.2025.101024 (PMC12172302; doi:10.1016/j.bbih.2025.101024)
Supplement: Multimedia component 1 [file mmc1.docx]

**Relationship between blood-cerebrospinal fluid barrier integrity, cardiometabolic, and inflammatory factors in schizophrenia-spectrum disorders**

Vladislav Yakimov^1,2,3#*^ (ORCID iD: 0000-0001-9559-7492), Iris Jäger^1*^, Lukas Roell^1,3^ (ORCID iD: 0000-0002-0284-2290), Emanuel Boudriot^1,4^ (ORCID iD: 0000-0001-6083-6318), Verena Meisinger^1^, Mattia Campana^1,5^, Lenka Krčmář^1,3^, Sean Halstead^6,7^ (ORCID iD: 0009-0000-4890-3506), Nicola Warren^6,7^ (ORCID iD: [0000-0002-0805-1182](https://orcid.org/0000-0002-0805-1182)), Dan Siskind^6,7^ (ORCID iD: [0000-0002-2072-9216](https://orcid.org/0000-0002-2072-9216)), Isabel Maurus^1,2^ (ORCID iD: 0000-0002-6208-5180), Alkomiet Hasan^8,9^, Peter Falkai^1,4,9^ (ORCID iD: 0000-0003-2873-8667), Andrea Schmitt^1,9,10^ (ORCID iD: 0000-0002-5426-4023), Florian J. Raabe^1,4,9^ (ORCID iD: 0000-0001-8538-0783)), Daniel Keeser^1,3^ (ORCID iD): 0000-0002-0244-1024), CDP-Working Group^1,2,3,4,5,8,9,10,11^, Elias Wagner^8,11*^, Joanna Moussiopoulou^1,3*^ (ORCID iD: 0000-0002-0157-6197)

^1^Department of Psychiatry and Psychotherapy, LMU University Hospital, LMU Munich, Munich, Germany

^2^International Max Planck Research School for Translational Psychiatry (IMPRS-TP), 80804 Munich, Germany

^3^NeuroImaging Core Unit Munich (NICUM), LMU University Hospital, LMU Munich, 80336 Munich, Germany

^4^Max Planck Institute of Psychiatry, 80804 Munich, Germany

^5^Department of Psychiatry and Psychotherapy, Medical Faculty, LVR Hospital of the Heinrich Heine University Düsseldorf, Düsseldorf, Germany

^6^Medical School, The University of Queensland, Brisbane, QLD, Australia

^7^Metro South Addiction and Mental Health, Brisbane, QLD, Australia

^8^Department of Psychiatry, Psychotherapy and Psychosomatics, Faculty of Medicine, University of Augsburg, 86156 Augsburg, Germany

^9^German Center for Mental Health (DZPG), partner site Munich/Augsburg, Germany

^10^Laboratory of Neuroscience (LIM27), Institute of Psychiatry, University of Sao Paulo, São Paulo, Brazil

^11^Evidence-based Psychiatry and Psychotherapy, Faculty of Medicine, University of Augsburg, Stenglinstrasse 2, 86156 Augsburg, Germany

* These authors contributed equally

**#Corresponding author:**

Dr. med. Vladislav Yakimov

Address: Department of Psychiatry and Psychotherapy, University Hospital, LMU Munich, Nussbaumstrasse 7, 80336 Munich, Germany

Email address: [V.Yakimov@med.uni-muenchen.de](mailto:V.Yakimov@med.uni-muenchen.de)

**Running title:** Blood-cerebrospinal fluid barrier disruption in schizophrenia

**Supplementary methods**

***Clinical assessments***

The clinical characterization was performed by trained study personnel as previously described by our working group^1,2^. The German version 7.0.2 of Mini International Neuropsychiatric Interview (M.I.N.I.)^3^, based on DSM-5 criteria, was conducted with all study participants to confirm the diagnosis. Symptom severity was assessed with the Positive and Negative Syndrome Scale (PANSS)^4^ and global functioning with the Global Assessment of Functioning (GAF)^5^ scale. The assessments were performed within four weeks around the lumbar puncture. Information regarding medication, duration of illness (DUI), BMI, blood pressure, heart rate, concomitant somatic conditions, and current smoking status was collected based on self-report and by examining medical reports. Current treatment or history of clozapine use was used as a proxy for treatment resistance, as previously suggested^6^.

To assess the cognitive performance of the participants, the Montreal Cognitive Assessment (MoCA)^7^ and the Trail-Making-Test (TMT, part A and B) were performed in a subgroup of participants. The MoCA assesses the following cognitive domains: short-term memory, visuospatial abilities, executive functions, attention, concentration, working memory, orientation, and language. It has been validated as a practical tool for measuring cognitive deficits in individuals with SSD^8^. The TMT covers the cognitive domains attention, visual search and scanning, processing speed, task switching, cognitive flexibility and executive function^9^, which are often affected in individuals with SSD.

***Blood and cerebrospinal fluid analyses***

In line with recommendations from the German schizophrenia guideline^10,11^, lumbar puncture was offered to all patients with first- (FEP) or multi-episode psychosis (MEP), who had not yet received CSF analysis in the past as part of the diagnostic work-up to exclude concurrent somatic etiologies. Paired CSF and serum samples were analyzed as part of the clinical routine diagnostics by the Institute of Laboratory Medicine, LMU Munich.

The CSF analysis (Table St1) included white blood cell counts (ref.: ≤ 5), total protein (ref.: 15-45 mg/dl), albumin (ref.: 0.1 – 0.3 g/l), and immunoglobulin G (IgG) (ref.: ≤ 0.034 g/l) levels as well as the presence of oligoclonal IgG bands (OCBs) and neuronal antibodies (incl., N-methyl-D-aspartate receptor (NMDA) antibodies, α-amino-3-hydroxy-5-methyl-4-isoxazolepropionic acid (AMPA) 1/2 receptor antibodies, dipeptidyl-peptidase-like protein 6 (DPPX) antibodies, contactin-associated protein-like 2 (CASPR2) antibodies, leucine-rich glioma-inactivated 1 (LGI1) antibodies, gamma-aminobutyric acid B receptor (GABA B) 1/2 antibodies).

Most of the study participants underwent a basic blood test, including full blood (N = 54) and serum (N = 52 – 56, depending on the variable assessed) analyses, within 3 weeks from the lumbar puncture as part of the clinical routine in our clinic. This was done during the morning hours under fasting conditions. The full blood analysis included a complete blood count and the serum analysis included assessment of C-reactive protein (CRP) (ref.: ≤ 0.5 mg/dl), triglycerides (ref.: ≤ 150 mg/dl), total cholesterol (ref.: ≤ 200 mg/dl), low-density lipoprotein (LDL) cholesterol (ref.: ≤ 116 mg/dl), high-density lipoprotein (HDL) cholesterol (ref.: > 40 mg/dl), glycated hemoglobin (HbA1c) (ref.: ≤ 5.7%), albumin (ref.: 3.5 – 5.2 g/dl), IgG levels (ref.: 7 – 16 g/l), and the presence of OCBs. Oligoclonal IgG bands in CSF and serum (collected at the same timepoint) were detected using a SEBIA HYDRASYS 2 SCAN FOCUSING semiautomated instrument according to the manufacturer`s instructions, which enables immunofixation and direct detection of OCBs on agarose gels using the HYDRAGEL 9CSF kit^10^. Cut-off values of CSF/serum albumin ratios (Q_alb_) were adjusted to age with the formula: Q_alb_ = (4 + age/15) × 10^–3 12^. To compute CSF/serum albumin and IgG ratios, serum and CSF were collected and assessed at the same timepoint. The neutrophil-to-lymphocyte ratio (NLR) and monocyte-to-lymphocyte ratio (MLR) were calculated by dividing the absolute number of neutrophils and monocytes each by the absolute number of lymphocytes per individual^13^.

***Magnetic resonance imaging***

A subset of 28 participants underwent brain magnetic resonance imaging (MRI) using a Siemens Magnetom Prisma 3T scanner (Siemens Healthineers AG, Erlangen, Germany) equipped with a 32-channel head coil. T1-weighted scans were acquired using a magnetization-prepared rapid gradient echo (MP-RAGE) sequence with an isotropic voxel size of 0.8 mm^3^, 208 slices, repetition time of 2500 ms, echo time of 2.22 ms, flip angle of 8°, and a field of view of 256 mm. Regional brain volumes, including the lateral ventricles, third and fourth ventricles were quantified in cubic millimeters using FreeSurfer software (version 7.3.2; <https://surfer.nmr.mgh.harvard.edu>)^14^. We utilized the FreeSurfer atlas to obtain the volumes of the left and right lateral ventricles, the third ventricle and the fourth ventricle. Additionally, the choroid plexus (ChP) in the lateral ventricles was manually segmented on the 3D-T1 images by one of the first authors (IJ), who was trained by a neuroimaging expert (DK). We employed ITK-SNAP software, version 4.2.0 (<http://www.itksnap.org>). The rater was blinded regarding clinical and imaging data and followed a previously published protocol for ChP segmentation^15^. The ChP as well as the ventricle measures were adjusted for total intracranial volume using the proportions method^16^.

Structural MRI data quality control was performed as previously described by our working group^17^. Specifically, a visual inspection and utilization of the quality control software MRIQC^18^ were conducted. Manual assessment included rating overall image quality and evaluating specific sequence-related metrics such as signal-to-noise ratio (SNR), contrast-to-noise ratio (CNR), coefficient of joint variation (CJV), foreground-background energy ratio (FBER), median intensity non-uniformity (INU). Images were flagged if they received poor manual quality ratings and/or showed at least one abnormal quality metric. After image processing, we visually inspected the FreeSurfer segmentations. No participants were excluded based on criteria related to insufficient image quality.

The structural isotropic 3D T1-weighted MR images were processed with FreeSurfer v7.2, employing *recon-all*^17^. It included motion correction and averaging^19^, non-brain tissue removal^20^, automated Talairach transformation, segmentation of subcortical white and grey matter volumes^14,21^, intensity normalization^22^, tessellation of the grey matter-white matter boundary, automated topology correction^23,24^, and surface deformation^25,26,27^. More information regarding the FreeSurfer pipeline is available at <http://surfer.nmr.mgh.harvard.edu/>.

***Statistical analyses***

The R language (v4.2.1, R Core Team, 2021) in RStudio environment (RStudio Team, 2020)^28^ was used for all statistical analyses and visualizations.

To investigate the relationships between BCB composite score and measures of psychopathology, cognition, or cerebroventricular regions, we computed Bayesian linear and logistic regression models, using the *brms* package^29^. Prior to conducting the Bayesian regression analyses, all continuous predictors and dependent variables were standardized (scaled to have a mean of zero and a standard deviation of one), using the *scale()* function in R. This standardization was performed to avoid artificial inflation of Bayes factors (BF_10_) due to large differences in variable units (e.g., HDL cholesterol and triglycerides).

The Bayes factor (BF_10_) expresses the degree to which the data support the alternative hypothesis (H_1_: effect size ≠ 0) relative to the null hypothesis (H_0_: effect size = 0). For instance, a BF_10_ of four indicates that the observed data are four times more likely under the alternative hypothesis than under the null hypothesis. In contrast to frequentist statistics, which relies on binary decisions based on a predefined significance threshold (e.g., *p* < 0.05), the BF_10_ offers a continuous measure of evidence and does not require correction for multiple comparisons^17^. Bayes factors (BF_10_) were interpreted following a commonly used heuristic classification scheme^32^: BF_10_ between 1 and 3 was considered anecdotal evidence, 3 to 10 moderate, 10 to 30 strong, 30 to 100 very strong, and > 100 extreme evidence in favor of the alternative hypothesis. A normal prior distribution with a mean of zero and a standard deviation of one was specified as a Bayesian prior for each predictor.

In the Bayesian linear regressions, BCB composite score, age, and sex were included as predictors, while the corresponding measures of psychopathology, cognition, and cerebroventricular regions served as dependent variables. In the models, including cognition measures, we used years of education as an additional predictor. To investigate the association between BCB composite score and treatment resistance, we computed a Bayesian logistic regression model including BCB composite score, age, sex, smoking status, and BMI as predictors and history of clozapine treatment as a dependent variable. To study the relationships between cardiometabolic risk factors (e.g., total cholesterol, HbA1c) or inflammatory measures (e.g., CRP) and BCB composite score, we computed Bayesian linear regressions, including the respective cardiometabolic or inflammatory measure, along age, sex, BMI and smoker status^30,31^ as independent variables and the BCB composite score as a dependent variable.

Complementary frequentist analyses were conducted using linear and logistic regression models, with statistical inference based on a significance threshold of *p* < 0.05. To investigate the relationships between BCB composite score (predictor variable) and measures of psychopathology, cognition, or cerebroventricular measures as outcome variables, we computed multiple linear regressions, controlling for age and sex as covariates. In the linear regression models, including cognition measures, we additionally controlled for years of education as a covariate. To investigate the association between BCB composite score (predictor variable) and treatment resistance (outcome variable), we computed a logistic regression controlling for age, sex, smoking status, and BMI as covariates. To study the relationships between cardiometabolic risk factors (e.g., total cholesterol, HbA1c) or inflammatory measures (e.g., CRP) as predictor variables and BCB composite score as outcome variable, we conducted multiple linear regressions controlling for age, sex, BMI, and smoker status, as previously suggested^30,31^. The threshold for statistical significance was set at *p* value < 0.05. Results from the descriptive statistics are shown as mean ± standard deviation (SD). We employed the Benjamini-Hochberg method^33^ for multiple testing correction within every group of sub-analyses. False discovery rate (FDR) adjusted *p-*values were reported as *q*-values.

Results from the descriptive statistics are shown as mean ± standard deviation (SD). Results from the Bayesian statistics are reported as scaled regression coefficients (β), along with their 95% credible intervals (95% CI) and Bayes factors (BF_10_).

**Figure Legends**

**Figure S1.** **Loadings Plot for Principal Component Analysis of BCB permeability markers.**

Loadings plot illustrating the projection of variables on the first two principal components from a Principal Component Analysis (PCA). The variables included in the analysis are total CSF protein, CSF/serum albumin ratio, and CSF/serum IgG ratio. The first principal component (PC1) is plotted on the x-axis, and the second principal component (PC2) is plotted on the y-axis. The size of the bubbles represents the magnitude of the loadings. N = 57. Abbreviations: BCB, blood-cerebrospinal fluid barrier; CSF, cerebrospinal fluid; PC1, first principal component; PC2, second principal component.

**Figure S2.**  **Relationship between blood-cerebrospinal fluid barrier integrity and measures of psychopathology as well as cognition.**

Regression plots illustrating associations between blood-cerebrospinal fluid barrier composite score, **(A)** PANSS positive, **(B)** PANSS negative, **(C)** PANSS general scores, **(D)** TMT-A, **(E)** TMT-B scores. Multiple Bayesian linear regressions were employed, controlling for age, sex and in (D) and (E) years of education. N = 47 in (A) – (C) and N = 34 in (D) and (E). Abbreviations: N, number of participants; BCB, blood-cerebrospinal fluid barrier; PANSS, Positive And Negative Syndrome Scale; TMT, Trail Making Test.

**Figure S3. Relationship between illness characteristics and blood-cerebrospinal fluid barrier integrity.**

Regression plots illustrating associations between **(A)** duration of illness and **(C)** duration of treatment and blood-cerebrospinal fluid barrier composite score. **(B)** Comparison of mean blood-cerebrospinal fluid barrier composite score between individuals with first-episode psychosis (red) and multiple-episode psychosis (turquoise) illustrated with box and violin plots. Multiple Bayesian linear regressions were employed, controlling for age and sex. N = 56 in (A), (C), and N = 57 in (B). Abbreviations: N, number of participants; BCB, blood-cerebrospinal fluid barrier; AP, antipsychotic; FEP, first-episode psychosis; MEP, multiple-episode psychosis.

**Figure S4. Relationship between blood-cerebrospinal fluid barrier integrity and cardiovascular factors.**

Regression plots illustrating associations between blood-cerebrospinal fluid barrier composite score, **(A)** glycated haemoglobin (HbA1c) and **(B)** systolic blood pressure. Two Bayesian linear regressions were employed, controlling for age, sex, BMI and smoking status. N = 52 in (A) and N = 55 in (B). Abbreviations: N, number of participants; BCB, blood-cerebrospinal fluid barrier; HbA1c, glycated haemoglobin; systolic BP, systolic blood pressure.

**Figure S5. Relationship between blood-cerebrospinal fluid barrier integrity and inflammatory markers.**

Regression plots illustrating associations between blood-cerebrospinal fluid barrier composite score, **(A)** absolute lymphocyte count and **(B)** C-reactive protein. Two Bayesian linear regressions were employed, controlling for age, sex, BMI and smoking status. N = 54. Abbreviations: N, number of participants; BCB, blood-cerebrospinal fluid barrier; C-reactive protein.

**Figure S6. Relationship between blood-cerebrospinal fluid barrier integrity and 3^rd^ or 4^th^ ventricle.**

Regression plots illustrating associations between blood-cerebrospinal fluid barrier composite score, **(A)** 3^rd^ ventricle volume and **(B)** 4^th^ ventricle volume. Multiple Bayesian linear regressions were employed, controlling for age and sex. N = 28. Abbreviations: N, number of participants; BCB, blood-cerebrospinal fluid barrier.

**Figures**

**Figure S1**

**
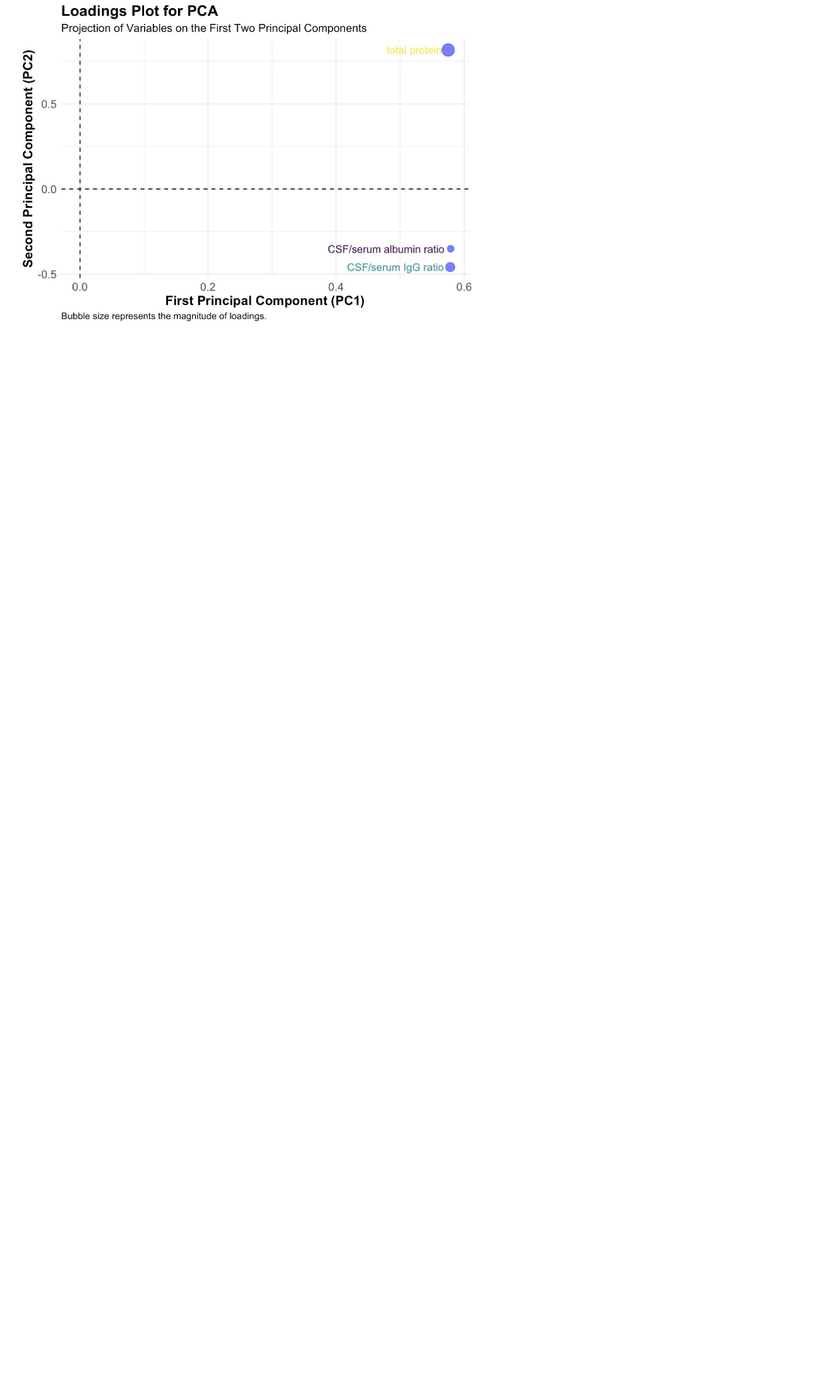
**

**Figure S2**


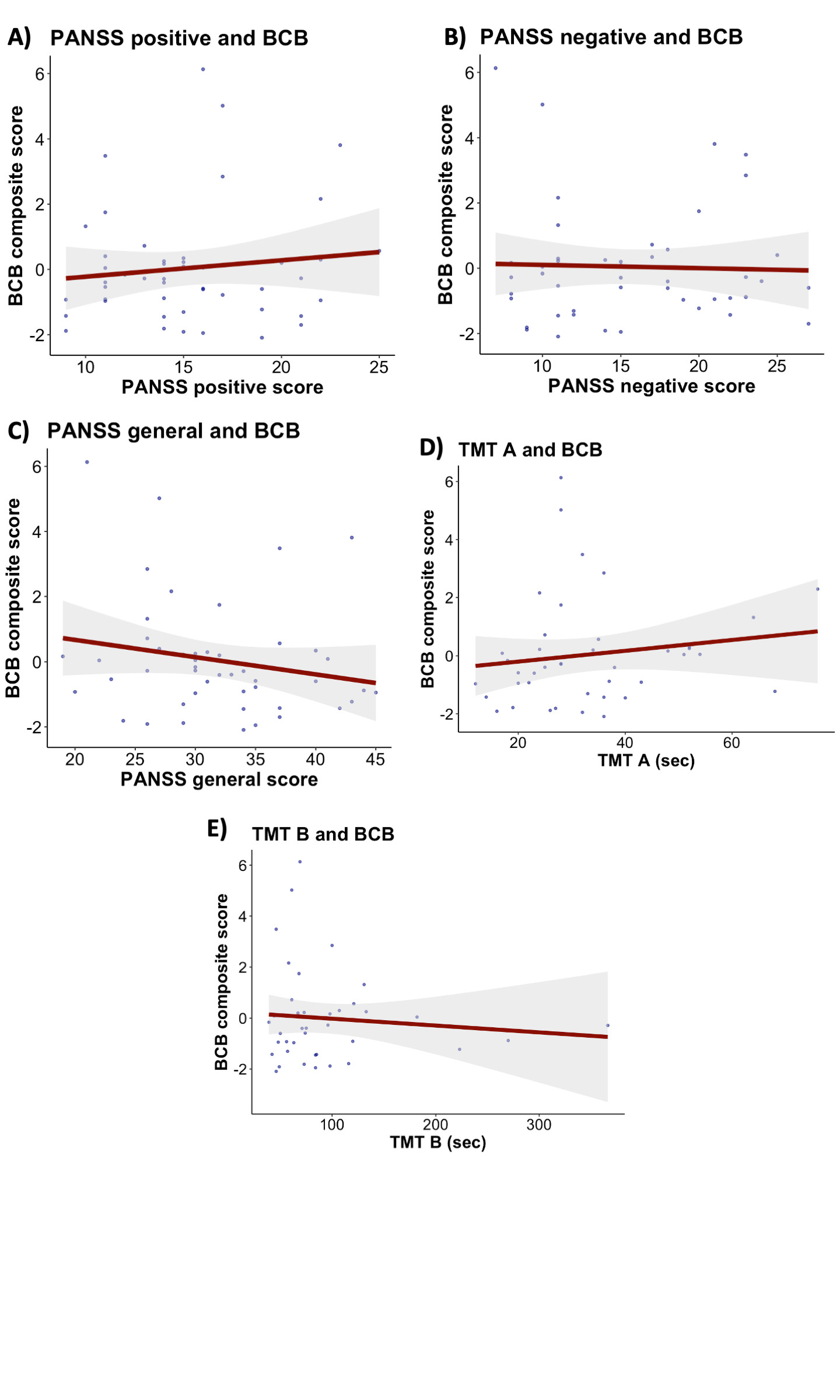


**Figure S3**

**
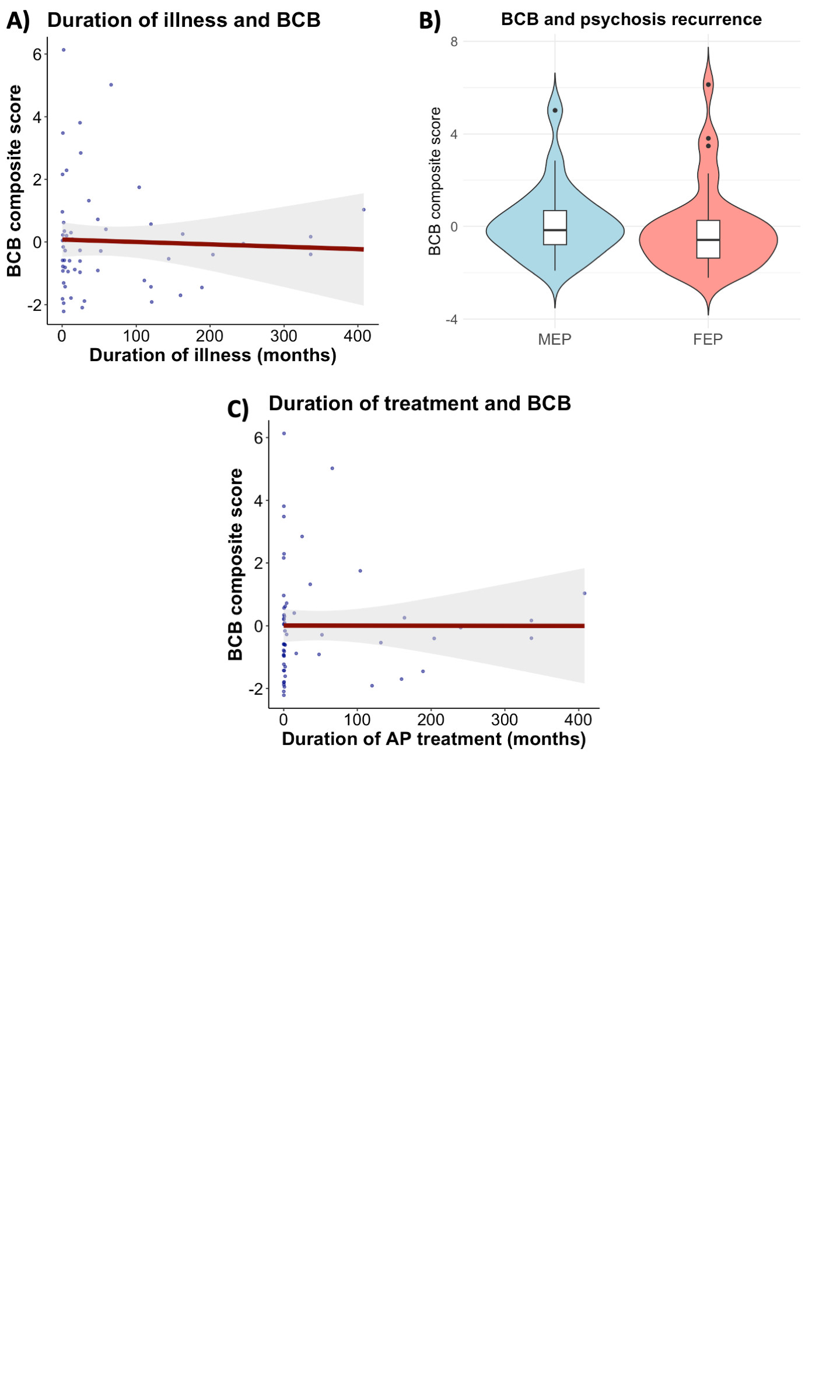
**

**Figure S4**

**
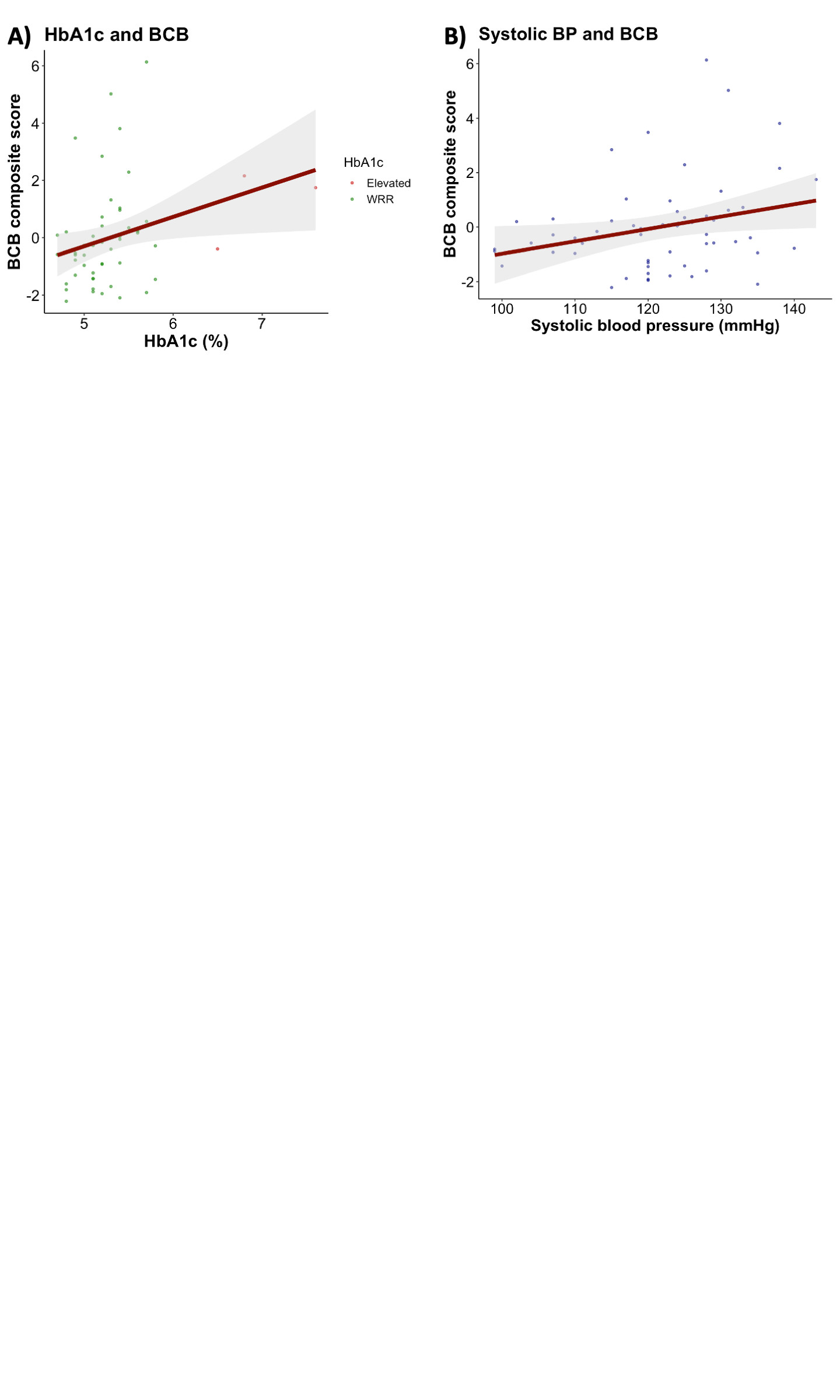
**

**Figure S5**

**
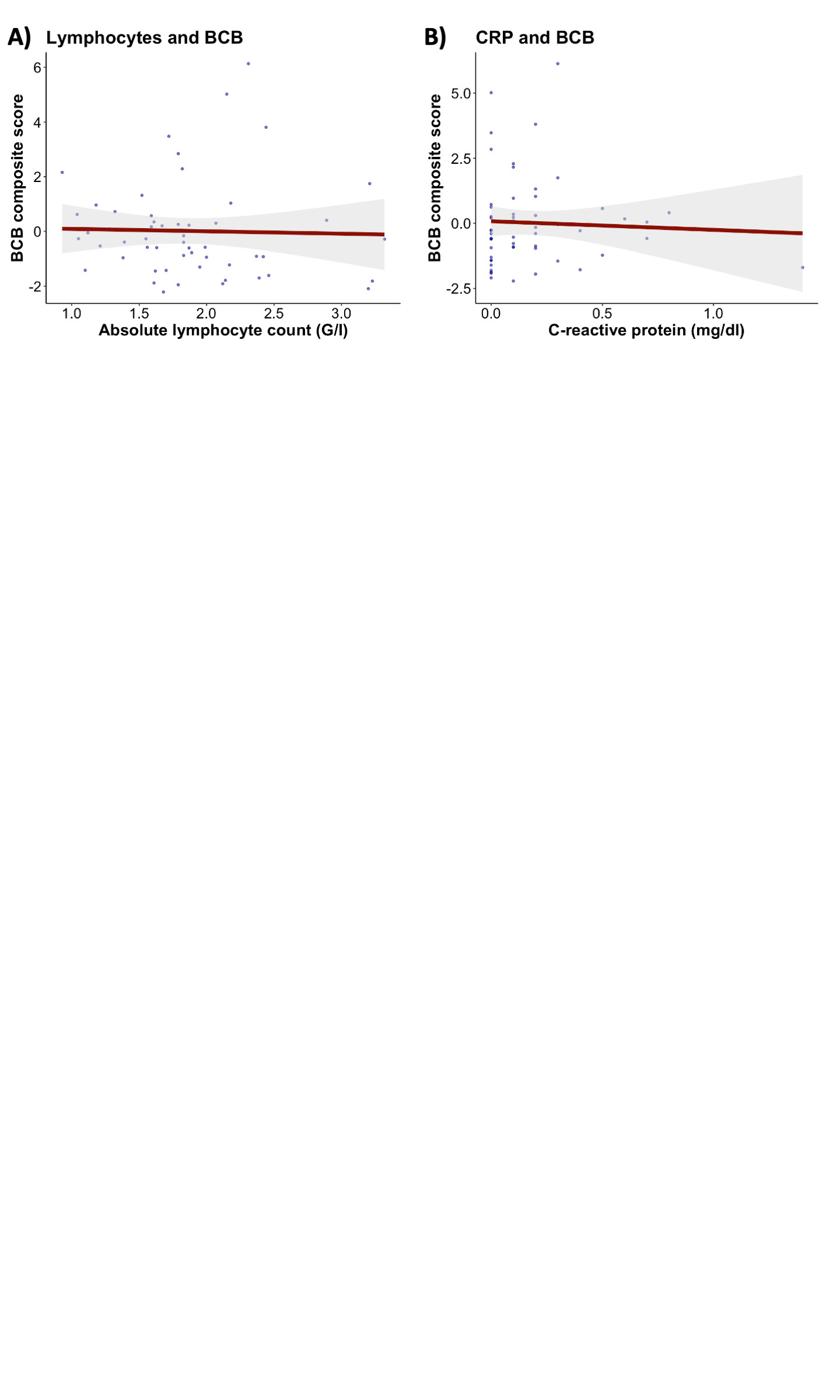
**

**Figure S6**

**
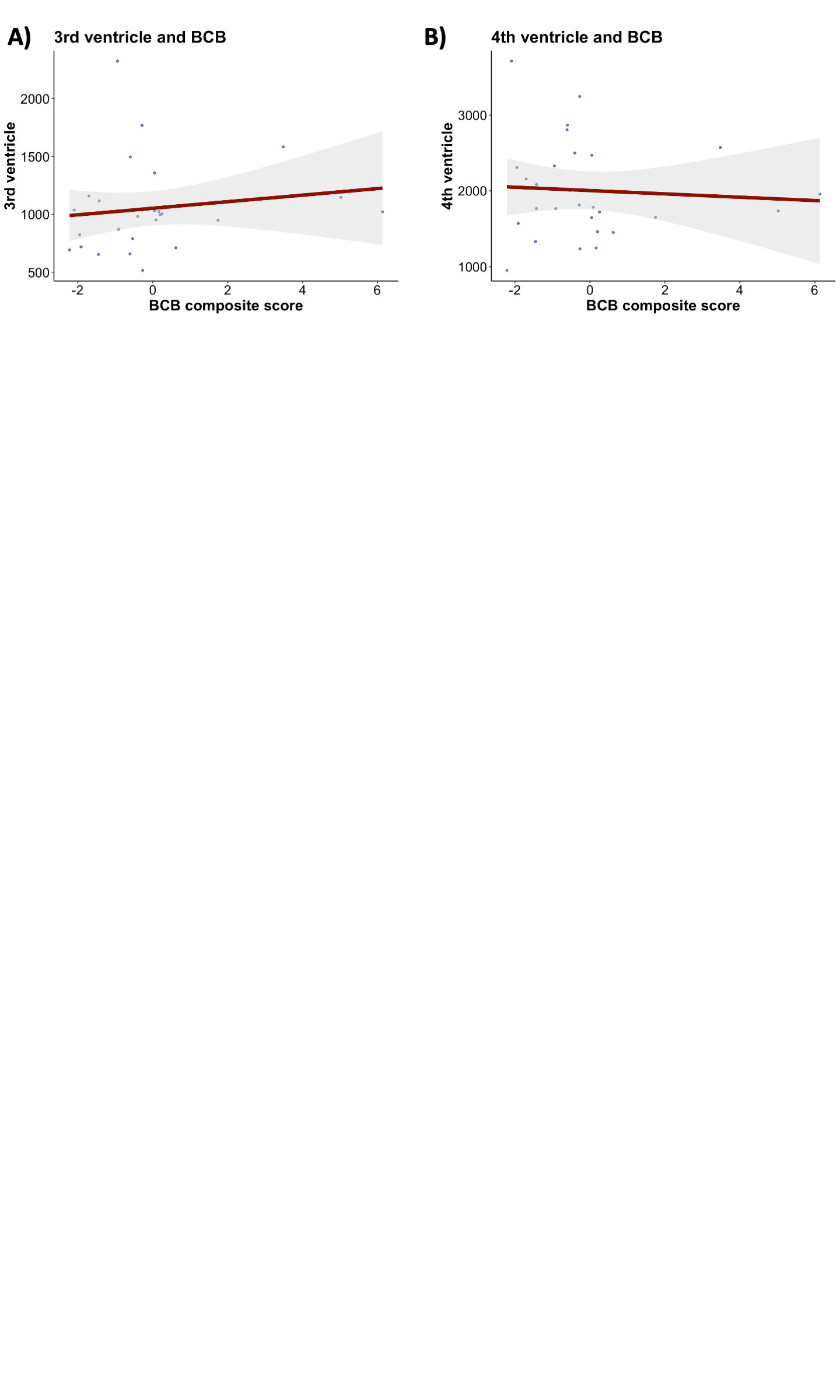
**

**Tables**

**Table St1 CSF parameter characteristics.**

| **CSF parameters** | **SSD**  *Mean* ± *SD*  *n (%)* | | | *N* |  |
| --- | --- | --- | --- | --- | --- |
| Protein level (mg/dl) | 42.42 ± 18.55 | | | 57 |  |
| Protein level elevated (yes:no) | 17:40 (29.8%) | | | 57 |  |
| CSF/serum albumin ratio | 6.53 ± 3.36 | | | 57 |  |
| CSF/serum albumin ratio elevated* (yes:no) | 24:33 (42.1%) | | | 57 |  |
| CSF/serum IgG ratio | 3.18 ± 1.66 | | | 57 |  |
| White Blood Cell Count (cells/µl) | 0.88 ± 1.09 | | | 57 |  |
| Pleocytosis (> 5/µl)) (yes:no) | 0:57 | | | 57 |  |
| OCBs (yes:no) | 5:52 (8.8%) | | | 57 |  |
| OCBs intrathecal synthesis (yes:no) | 0:57 | | | 57 |  |
| Neuronal autoantibodies** (yes:no) |  | 0:55 | 55 | | |

CSF, cerebrospinal fluid; *N*, number of participants; OCBs, oligoclonal IgG bands; *SD*, standard deviation

*CSF/serum albumin ratio (Qalb) cut-off is adjusted for age according to the formula Qalb = (4 + age/15) × 10^–3^

**neuronal antibodies include NMDA antibodies, Glutamate (AMPA 1/2) receptor antibodies, DPPX antibodies, CASPR2 antibodies, LGI1 antibodies, GABA B (1/2) antibodies

**Table St2 Blood parameter characteristics.**

| **Blood Parameters** | **Patients with SSD**  *Mean* ± *SD*  *n (%)* | *N* |
| --- | --- | --- |
| Total cholesterol (mg/dl) | 178.20 ± 32.64 | 53 |
| Total cholesterol elevated (yes:no) | 14:41 (25.5%) | 53 |
| HDL cholesterol (mg/dl) | 53.24 ± 16.48 | 53 |
| HDL cholesterol reduced (yes:no) | 27:28 (49.1%) | 53 |
| LDL cholesterol (mg/dl) | 109.10 ± 37.74 | 53 |
| LDL cholesterol elevated (yes:no) | 19:36 (34.5%) | 53 |
| Triglyceride (mg/dl) | 118.50 ± 82.99 | 53 |
| Triglyceride elevated (yes:no) | 14:41 (25.5%) | 53 |
| HbA1c (%) | 5.34 ± 0.51 | 52 |
| HbA1c elevated (yes:no) | 3:51 (5.6%) | 52 |
| Serum-CRP (mg/dl) | 0.18 ± 0.26 | 54 |
| Serum-CRP elevated (yes:no) | 5:51 (8.9%) | 54 |
| Neutrophiles (thou./µl) | 4.27 ± 1.82 | 54 |
| Neutrophiles elevated (yes:no) | 3:53 (5.4%) | 54 |
| Monocytes (thou./µl) | 0.53 ± 0.17 | 54 |
| Monocytes elevated (yes:no) | 3:53 (5.4%) | 54 |
| Lymphocytes (thou./µl) | 1.86 ± 0.57 | 54 |
| Lymphocytes elevated (yes:no) | 0:56 (0%) | 54 |
| NLR | 2.43 ± 1.03 | 54 |
| MLR | 0.30 ± 0.10 | 54 |

MLR, Monocyte-Lymphocyte-Ratio; *N*, number of participants; NLR, Neutrophile-Lymphocyte-Ratio; *SD*, standard deviation

**Table St3 Concurrent medication.**

|  | **Patients with SSD** |  |
| --- | --- | --- |
| Medication (current) | *N* (%) |  |
| Antipsychotics | 48 (84.2%) |  |
| Amisulpride  Aripiprazole  Cariprazine  Clozapine  Haloperidole  Olanzapine  Quetiapine  Risperidone/Paliperidone | 11 (19.3%)  5 (8.8%)  2 (3.5%)  4 (7.0%)  5 (8.8%)  15 (26.3%)  10 (17.5%)  15 (26.3%) |  |
| Benzodiazepines | 17 (29.8%) |  |
| Lorazepam  Diazepam | 16 (28.1%)  1 (1.8%) |  |
| Antidepressants | 11 (19.3%) |  |
| Duloxetine  Mirtazapine  Sertraline  Trazodone  Trimipramine  Venlafaxine | 1 (1.8%)  3 (5.3%)  4 (7.0%)  3 (5.3%)  1 (1.8%)  3 (5.3%) |  |
|  |  |  |

*N*, number of participants; *SD*, standard deviation; SSD, Schizophrenia Spectrum Disorder

| **List of Abbreviations** | |
| --- | --- |
| Abbreviation | Explanation |
| BC | blood count |
| BCB | blood-cerebrospinal fluid-barrier |
| BMI | body-mass-index |
| BF10 | Bayes factor |
| CI | confidence interval/credible interval |
| CRP | C-reactive protein |
| CSF | cerebrospinal fluid |
| DUI | duration of illness |
| FEP | first episode psychosis |
| GAF | Global Assessment of Functioning |
| HDL | high-density lipoprotein |
| LDL | low-density lipoprotein |
| LL | lower limit |
| MLR | monocyte-to-lymphocyte ratio |
| MoCA | Montreal Cognitive Assessment |
| N | number of participants |
| NLR | neutrophile-to-lymphocyte ratio |
| p | p-value |
| PANSS | Positive And Negative Syndrome Scale |
| q | false dicovery rate adjustet p-value |
| SSD | schizophrenia-spectrum disorder |
| SZ | schizophrenia |
| SZA | schizoaffective disorder |
| TMT | Trail-Making Test |
| UL | upper limit |

| **Table S1 Complete parameter estimates – association analyses of general disease characteristic measures and blood-CSF barrier (BCB) composite score in SSD** | | | | | | | |
| --- | --- | --- | --- | --- | --- | --- | --- |
|  |  |  |  |  |  | |  |
| **Table S1.1 Association between duration of illness (DUI) and BCB composite score** | | | |  |  | |  |
| Response | Predictor | Estimate | 95% CI [LL, UL] | N | *p* | | *q* |
| BCB composite score | (Intercept) | -1.404 | [-2.586, -0.022] | 56 | 0.052 | | 0.078 |
| BCB composite score | DUI (months) | -0.003 | [-0.007, 0.001] | 56 | 0.25 | | 0.519 |
| BCB composite score | age | 0.05 | [0.014 , 0.085] | 56 | 0.022* | | 0.033* |
| BCB composite score | sex | -0.396 | [-1.270, 0.477] | 56 | 0.451 | | 0.451 |
|  |  |  |  |  |  | |  |
| **Table S1.2 Association between duration of antipsychotic treatment and BCB composite score** | | | | |  | |  |
| Response | Predictor | Estimate | 95% CI [LL, UL] | N | *p* | | *q* |
| BCB composite score | (Intercept) | -1.504 | [-2.694, -0.315] | 56 | 0.039* | | 0.078 |
| BCB composite score | Duration AP treatment (months) | -0.002 | [-0.007, 0.002] | 56 | 0.346 | | 0.519 |
| BCB composite score | age | 0.051 | [0.015, 0.086] | 56 | 0.021* | | 0.033* |
| BCB composite score | sex | -0.442 | [-1.332, 0.448] | 56 | 0.409 | | 0.451 |
|  |  |  |  |  |  | |  |
| **Table S1.3 Association between FEP status and BCB composite score** | | | |  |  | |  |
| Response | Predictor | Estimate | 95% CI [LL, UL] | N | *p* | | *q* |
| BCB composite score | (Intercept) | -1.177 | [-2.422, 0.069] | 57 | 0.12 | | 0.12 |
| BCB composite score | FEP status | -0.287 | [-1.055, 0.482] | 57 | 0.535 | | 0.535 |
| BCB composite score | age | 0.042 | [0.010, 0.075] | 57 | 0.034* | | 0.034* |
| BCB composite score | sex | -0.378 | [-1.254, 0.498] | 57 | 0.473 | | 0.102 |
| **Table S1.4 Bayesian test statistics** | | | |  |  |  |  |
| **Response** | **Predictor** | **Estimate** | **95% CI [LL, UL]** | ***BF10*** | |  |  |
| BCB composite score | DUI (months) | -0.257 | [-0.736, 0.226] | 0.435 | |  |  |
| BCB composite score | Duration AP treatment (months) | -0.204 | [-0.689, 0.274] | 0.334 | |  |  |
| BCB composite score | FEP status | -0.238 | [-1.062, 0.588] | 0.498 | |  |  |

| **Table S2 Complete parameter estimates – association analyses of disease severity measures and blood-CSF barrier (BCB) composite score in SSD** | | | | | | | |
| --- | --- | --- | --- | --- | --- | --- | --- |
|  |  |  |  |  |  | |  |
| **Table S2.1 Association between BCB composite score and GAF score** | | | |  |  | |  |
| Response | Predictor | Estimate | 95% CI [LL, UL] | N | *p* | | *q* |
| GAF | (Intercept) | 42.919 | [33.155, 52.684] | 47 | <0.001*** | | <0.001*** |
| GAF | BCB composite score | 0.604 | [-1.026, 2.234] | 47 | 0.537 | | 0.806 |
| GAF | age | 0.166 | [-0.124, 0.455] | 47 | 0.341 | | 0.409 |
| GAF | sex | -5.927 | [-12.414, 0.560] | 47 | 0.132 | | 0.163 |
|  |  |  |  |  |  | |  |
| **Table S2.2 Association between BCB composite score and treatment resistance** | | | | |  | |  |
| Response | Predictor | Estimate | 95% CI [LL, UL] | N | *p* | | *q* |
| Clozapine lifetime | (Intercept) | -9.63 | [-17.454, -1.799] | 55 | 0.043* | | 0.043* |
| Clozapine lifetime | BCB composite score | 0.668 | [0.158, 1.177] | 55 | 0.031* | | 0.186 |
| Clozapine lifetime | age | 0.028 | [-0.044, 0.101] | 55 | 0.518 | | 0.583 |
| Clozapine lifetime | sex | -1.817 | [-4.683, 1.050] | 55 | 0.297 | | 0.297 |
| Clozapine lifetime | smoking status | 0.065 | [-1.677, 1.806] | 55 | 0.951 | | 0.951 |
| Clozapine lifetime | BMI | 0.233 | [-0.018, 0.483] | 55 | 0.126 | | 0.126 |
|  |  |  |  |  |  | |  |
| **Table S2.3 Association between BCB composite score and PANSS total score** | | | | |  | |  |
| Response | Predictor | Estimate | 95% CI [LL, UL] | N | *p* | | *q* |
| PANSS total score | (Intercept) | 68.26 | [57.592, 78.928] | 47 | <0.001*** | | <0.001*** |
| PANSS total score | BCB composite score | -0.153 | [-2.000, 1.694] | 47 | 0.89 | | 0.89 |
| PANSS total score | age | -0.237 | [-0.561, 0.086] | 47 | 0.225 | | 0.396 |
| PANSS total score | sex | 8.698 | [1.306, 16.089] | 47 | 0.054 | | 0.163 |
|  |  |  |  |  |  | |  |
| **Table S2.4 Association between BCB composite score and PANSS positive score** | | | | |  | |  |
| Response | Predictor | Estimate | 95% CI [LL, UL] | N | *p* | | *q* |
| PANSS positive score | (Intercept) | 13.627 | [10.231, 17.023] | 47 | <0.001*** | | <0.001*** |
| PANSS positive score | BCB composite score | 0.255 | [-0.333, 0.843] | 47 | 0.47 | | 0.806 |
| PANSS positive score | age | 0.034 | [-0.069, 0.137] | 47 | 0.583 | | 0.583 |
| PANSS positive score | sex | 2.129 | [-0.224, 4.482] | 47 | 0.136 | | 0.163 |
|  |  |  |  |  |  | |  |
| **Table S2.5 Association between BCB composite score and PANSS negative score** | | | | |  | |  |
| Response | Predictor | Estimate | 95% CI [LL, UL] | N | *p* | | *q* |
| PANSS negative score | (Intercept) | 20.086 | [15.440, 24.732] | 47 | <0.001*** | | <0.001*** |
| PANSS negative score | BCB composite score | 0.157 | [-0.647, 0.961] | 47 | 0.744 | | 0.89 |
| PANSS negative score | age | -0.165 | [-0.306, -0.024] | 47 | 0.055 | | 0.33 |
| PANSS negative score | sex | 3.364 | [0.145, 6.583] | 47 | 0.086 | | 0.163 |
|  |  |  |  |  |  | |  |
| **Table S2.6 Association between BCB composite score and PANSS general score** | | | | |  | |  |
| Response | Predictor | Estimate | 95% CI [LL, UL] | N | *p* | | *q* |
| PANSS general score | (Intercept) | 34.506 | [29.159, 39.854] | 47 | <0.001*** | | <0.001*** |
| PANSS general score | BCB composite score | -0.53 | [-1.456, 0.396] | 47 | 0.341 | | 0.806 |
| PANSS general score | age | -0.109 | [-0.271, 0.053] | 47 | 0.264 | | 0.396 |
| PANSS general score | sex | 3.383 | [-0.323, 7.088] | 47 | 0.132 | | 0.163 |
| **Table S2.7 Bayesian test statistics** | | | |  |  |  |  |
| Response | Predictor | Estimate | 95% CI [LL, UL] | *BF10* | |  |  |
| GAF | BCB composite score | 0.168 | [-0.358, 0.686] | 0.317 | |  |  |
| Clozapine lifetime | BCB composite score | 1.18 | [-0.005, 2.342] | 4.127 | |  |  |
| PANSS total score | BCB composite score | -0.058 | [-0.580, 0.466] | 0.272 | |  |  |
| PANSS positive score | BCB composite score | 0.175 | [-0.351, 0.706] | 0.342 | |  |  |
| PANSS negative score | BCB composite score | 0.06 | [-0.480, 0.589] | 0.276 | |  |  |
| PANSS general score | BCB composite score | -0.258 | [-0.780, 0.260] | 0.422 | |  |  |

| **Table S3 Complete parameter estimates – association analyses of cognitive measures and blood-CSF barrier (BCB) composite score in SSD** | | | | | | | |
| --- | --- | --- | --- | --- | --- | --- | --- |
|  |  |  |  |  |  | |  |
| **Table S3.1 Association between BCB composite score and TMT A time** | | | |  |  | |  |
| Response | Predictor | Estimate | 95% CI [LL, UL] | N | *p* | | *q* |
| TMT A time | (Intercept) | 42.571 | [23.891, 61.250] | 34 | 0.001*** | | 0.002** |
| TMT A time | BCB composite score | -0.527 | [-2.620, 1.566] | 34 | 0.672 | | 0.672 |
| TMT A time | age | 0.552 | [0.131, 0.973] | 34 | 0.034* | | 0.102 |
| TMT A time | sex | -3.983 | [-13.937, 5.972] | 34 | 0.502 | | 0.864 |
| TMT A time | education (years) | -1.786 | [-2.882, -0.690] | 34 | 0.01** | | 0.015* |
|  |  |  |  |  |  | |  |
| **Table S3.2 Association between BCB composite score and TMT B time** | | | |  |  | |  |
| Response | Predictor | Estimate | 95% CI [LL, UL] | N | *p* | | *q* |
| TMT B time | (Intercept) | 143.756 | [53.514, 233.999] | 34 | 0.011* | | 0.011* |
| TMT B time | BCB composite score | -5.215 | [-15.328,  4.898] | 34 | 0.388 | | 0.672 |
| TMT B time | age | 0.973 | [-1.060, 3.006] | 34 | 0.423 | | 0.423 |
| TMT B time | sex | -4.759 | [-52.851, 43.333] | 34 | 0.868 | | 0.868 |
| TMT B time | education (years) | -5.37 | [-10.667, -0.074] | 34 | 0.096 | | 0.096 |
|  |  |  |  |  |  | |  |
| **Table S3.3 Association between BCB composite score and MoCA score** | | | |  |  | |  |
| Response | Predictor | Estimate | 95% CI [LL, UL] | N | *p* | | *q* |
| MoCA score | (Intercept) | 18.786 | [14.282, 23.290] | 35 | <0.001*** | | <0.001*** |
| MoCA score | BCB composite score | 0.185 | [-0.319, 0.689] | 35 | 0.538 | | 0.672 |
| MoCA score | age | -0.078 | [-0.179, 0.023] | 35 | 0.2 | | 0.3 |
| MoCA score | sex | -0.799 | [-3.196, 1.599] | 35 | 0.576 | | 0.864 |
| MoCA score | education (years) | 0.623 | [0.361, 0.886] | 35 | <0.001*** | | 0.001*** |
| **Table S3.4 Bayesian test statistics** | | | |  |  |  |  |
| Response | Predictor | Estimate | 95% CI [LL, UL] | *BF10* | |  |  |
| TMT A time | BCB composite score | -0.077 | [-0.822, 0.683] | 0.381 | |  |  |
| TMT B time | BCB composite score | -0.253 | [-0.936, 0.446] | 0.439 | |  |  |
| MoCA score | BCB composite score | 0.16 | [-0.583, 0.891] | 0.403 | |  |  |

| **Table S4 Complete parameter estimates – association analyses of cardiometabolic factors and blood-CSF barrier (BCB) composite score in SSD** | | | | | | | |
| --- | --- | --- | --- | --- | --- | --- | --- |
|  |  |  |  |  |  | |  |
| **Table S4.1 Association between BCB composite score and total cholesterol** | | | | |  | |  |
| Response | Predictor | Estimate | 95% CI [LL, UL] | N | *p* | | *q* |
| BCB composite score | (Intercept) | -2.03 | [-4.511, 0.452] | 53 | 0.177 | | 0.212 |
| BCB composite score | total cholesterol | 0.026 | [0.014, 0.038] | 53 | 0.001** | | 0.001** |
| BCB composite score | age | 0.031 | [-0.002, 0.064] | 53 | 0.121 | | 0.145 |
| BCB composite score | sex | -0.381 | [-1.175, 0.412] | 53 | 0.424 | | 0.761 |
| BCB composite score | smoker status | 0.679 | [-0.010, 1.368] | 53 | 0.105 | | 0.152 |
| BCB composite score | BMI | -0.147 | [-0.237, -0.056] | 53 | 0.009** | | 0.022* |
|  |  |  |  |  |  | |  |
| **Table S4.2 Association between BCB composite score and LDL cholesterol** | | | | |  | |  |
| Response | Predictor | Estimate | 95% CI [LL, UL] | N | *p* | | *q* |
| BCB composite score | (Intercept) | -0.006 | [-2.293, 2.282] | 53 | 0.997 | | 0.997 |
| BCB composite score | LDL cholesterol | 0.023 | [0.013, 0.033] | 53 | <0.001*** | | 0.001** |
| BCB composite score | age | 0.027 | [-0.006, 0.060] | 53 | 0.182 | | 0.182 |
| BCB composite score | sex | -0.236 | [-1.026, 0.553] | 53 | 0.618 | | 0.761 |
| BCB composite score | smoker status | 0.807 | [0.124, 1.489] | 53 | 0.053 | | 0.152 |
| BCB composite score | BMI | -0.14 | [-0.229, -0.051] | 53 | 0.011* | | 0.022* |
|  |  |  |  |  |  | |  |
| **Table S4.3 Association between BCB composite score and HDL cholesterol** | | | | |  | |  |
| Response | Predictor | Estimate | 95% CI [LL, UL] | N | *p* | | *q* |
| BCB composite score | (Intercept) | 3.579 | [0.619, 6.538] | 53 | 0.048* | | 0.212 |
| BCB composite score | HDL cholesterol | -0.045 | [-0.067, -0.022] | 53 | 0.002** | | 0.003** |
| BCB composite score | age | 0.053 | [0.021,  0.085] | 53 | 0.008 | | 0.024* |
| BCB composite score | sex | -0.02 | [-0.842, 0.802] | 53 | 0.968 | | 0.968 |
| BCB composite score | smoker status | 0.613 | [-0.094, 1.321] | 53 | 0.152 | | 0.152 |
| BCB composite score | BMI | -0.123 | [-0.212, -0.033] | 53 | 0.026* | | 0.039* |
|  |  |  |  |  |  | |  |
| **Table S4.4 Association between BCB composite score and triglycerides** | | | | |  | |  |
| Response | Predictor | Estimate | 95% CI [LL, UL] | N | *p* | | *q* |
| BCB composite score | (Intercept) | 1.803 | [-0.393, 4.000] | 53 | 0.175 | | 0.212 |
| BCB composite score | Triglycerides | 0.013 | [0.008, 0.017] | 53 | <0.001*** | | <0.001*** |
| BCB composite score | age | 0.058 | [0.029, 0.087] | 53 | 0.002** | | 0.012* |
| BCB composite score | sex | -0.218 | [-0.947, 0.511] | 53 | 0.619 | | 0.761 |
| BCB composite score | smoker status | 0.666 | [0.034, 1.297] | 53 | 0.084 | | 0.152 |
| BCB composite score | BMI | -0.209 | [-0.299, -0.120] | 53 | <0.001*** | | 0.002** |
|  |  |  |  |  |  | |  |
| **Table S4.5 Association between BCB composite score and systolic blood pressure** | | | | |  | |  |
| Response | Predictor | Estimate | 95% CI [LL, UL] | N | *p* | | *q* |
| BCB composite score | (Intercept) | -4.027 | [-8.622, 0.567] | 55 | 0.148 | | 0.212 |
| BCB composite score | systolic BP | 0.037 | [0.001, 0.073] | 55 | 0.088 | | 0.105 |
| BCB composite score | age | 0.04 | [0.007, 0.074] | 55 | 0.05* | | 0.077 |
| BCB composite score | sex | -0.249 | [-1.121, 0.623] | 55 | 0.634 | | 0.761 |
| BCB composite score | smoker status | 0.656 | [-0.082, 1.395] | 55 | 0.143 | | 0.152 |
| BCB composite score | BMI | -0.081 | [-0.174, 0.011] | 55 | 0.146 | | 0.146 |
|  |  |  |  |  |  | |  |
| **Table S4.6 Association between BCB composite score and HbA1c** | | | |  |  | |  |
| Response | Predictor | Estimate | 95% CI [LL, UL] | N | *p* | | *q* |
| BCB composite score | (Intercept) | -3.441 | [-7.607, 0.726] | 52 | 0.172 | | 0.212 |
| BCB composite score | HbA1c | 0.819 | [-0.012, 1.650] | 52 | 0.105 | | 0.105 |
| BCB composite score | age | 0.044 | [0.007, 0.080] | 52 | 0.051 | | 0.077 |
| BCB composite score | sex | -0.381 | [-1.258, 0.496] | 52 | 0.469 | | 0.761 |
| BCB composite score | smoker status | 0.818 | [0.052, 1.583] | 52 | 0.08 | | 0.152 |
| BCB composite score | BMI | -0.1 | [-0.198, -0.002] | 52 | 0.095 | | 0.114 |
| **Table S4.7 Bayesian test statistics** | | | |  |  |  |  |
| Response | Predictor | Estimate | 95% CI [LL, UL] | *BF10* | |  |  |
| BCB composite score | total cholesterol | 0.807 | [0.350, 1.262] | 126.1 | |  |  |
| BCB composite score | LDL cholesterol | 0.812 | [0.376, 1.25] | 1084 | |  |  |
| BCB composite score | HDL cholesterol | -0.703 | [-1.132, -0.269] | 33.3 | |  |  |
| BCB composite score | Triglycerides | 0.992 | [0.577, 1.406] | 10103 | |  |  |
| BCB composite score | systolic BP | 0.381 | [-0.055, 0.817] | 0.913 | |  |  |
| BCB composite score | HbA1c | 0.401 | [-0.094, 0.894] | 0.952 | |  |  |

| **Table S5 Complete parameter estimates – association analyses of peripheral inflammatory markers and blood-CSF barrier (BCB) composite score in SSD** | | | | | | | |
| --- | --- | --- | --- | --- | --- | --- | --- |
|  |  |  |  |  |  | |  |
| **Table S5.1 Association between BCB composite score and absolute neutrophil count** | | | | |  | |  |
| Response | Predictor | Estimate | 95% CI [LL, UL] | N | *p* | | *q* |
| BCB composite score | (Intercept) | 0.238 | [-2.329, 2.805] | 54 | 0.87 | | 1 |
| BCB composite score | BC neutrophil granulocytes | 0.136 | [-0.095, 0.367] | 54 | 0.328 | | 0.949 |
| BCB composite score | age | 0.045 | [0.011, 0.079] | 54 | 0.033* | | 0.039* |
| BCB composite score | sex | -0.529 | [-1.431, 0.373] | 54 | 0.331 | | 0.443 |
| BCB composite score | smoker status | 0.575 | [-0.234, 1.383] | 54 | 0.239 | | 0.271 |
| BCB composite score | BMI | -0.093 | [-0.191, 0.006] | 54 | 0.122 | | 0.21 |
|  |  |  |  |  |  | |  |
| **Table S5.2 Association between BCB composite score and absolute monocyte count** | | | | |  | |  |
| Response | Predictor | Estimate | 95% CI [LL, UL] | N | *p* | | *q* |
| BCB composite score | (Intercept) | 0 | [-2.651, 2.650] | 54 | 1 | | 1 |
| BCB composite score | BC monocytes | 1.25 | [-1.287, 3.787] | 54 | 0.413 | | 0.949 |
| BCB composite score | age | 0.045 | [0.011, 0.079] | 54 | 0.031* | | 0.039* |
| BCB composite score | sex | -0.492 | [-1.389, 0.406] | 54 | 0.363 | | 0.443 |
| BCB composite score | smoker status | 0.559 | [-0.282, 1.400] | 54 | 0.271 | | 0.271 |
| BCB composite score | BMI | -0.088 | [-0.186, 0.010] | 54 | 0.138 | | 0.21 |
|  |  |  |  |  |  | |  |
| **Table S5.3 Association between BCB composite score and absolute lymphocyte count** | | | | |  | |  |
| Response | Predictor | Estimate | 95% CI [LL, UL] | N | *p* | | *q* |
| BCB composite score | (Intercept) | 0.303 | [-2.315, 2.920] | 54 | 0.847 | | 1 |
| BCB composite score | BC lymphocytes | 0.03 | [-0.755, 0.816] | 54 | 0.949 | | 0.949 |
| BCB composite score | age | 0.046 | [0.011, 0.081] | 54 | 0.031* | | 0.039* |
| BCB composite score | sex | -0.426 | [-1.336, 0.485] | 54 | 0.437 | | 0.443 |
| BCB composite score | smoker status | 0.721 | [-0.085, 1.528] | 54 | 0.14 | | 0.21 |
| BCB composite score | BMI | -0.081 | [-0.189, 0.026] | 54 | 0.209 | | 0.21 |
|  |  |  |  |  |  | |  |
| **Table S5.4 Association between BCB composite score and NLR** | | |  |  |  | |  |
| Response | Predictor | Estimate | 95% CI [LL, UL] | N | *p* | | *q* |
| BCB composite score | (Intercept) | 0.002 | [-2.755, 2.759] | 54 | 0.999 | | 1 |
| BCB composite score | NLR | 0.133 | [-0.276, 0.541] | 54 | 0.588 | | 0.949 |
| BCB composite score | age | 0.044 | [0.009, 0.079] | 54 | 0.039* | | 0.039* |
| BCB composite score | sex | -0.429 | [-1.318, 0.460] | 54 | 0.423 | | 0.443 |
| BCB composite score | smoker status | 0.705 | [-0.068, 1.478] | 54 | 0.132 | | 0.21 |
| BCB composite score | BMI | -0.076 | [-0.174, 0.021] | 54 | 0.195 | | 0.21 |
|  |  |  |  |  |  | |  |
| **Table S5.5 Association between BCB composite score and MLR** | | |  |  |  | |  |
| Response | Predictor | Estimate | 95% CI [LL, UL] | N | *p* | | *q* |
| BCB composite score | (Intercept) | 0.035 | [-2.996, 3.066] | 54 | 0.984 | | 1 |
| BCB composite score | MLR | 0.745 | [-3.425, 4.914] | 54 | 0.766 | | 0.949 |
| BCB composite score | age | 0.045 | [0.010, 0.080] | 54 | 0.035* | | 0.039* |
| BCB composite score | sex | -0.411 | [-1.303, 0.480] | 54 | 0.443 | | 0.443 |
| BCB composite score | smoker status | 0.705 | [-0.079, 1.489] | 54 | 0.138 | | 0.21 |
| BCB composite score | BMI | -0.076 | [-0.175, 0.024] | 54 | 0.209 | | 0.21 |
|  |  |  |  |  |  | |  |
| **Table S5.6 Association between BCB composite score and CRP** | | |  |  |  | |  |
| Response | Predictor | Estimate | 95% CI [LL, UL] | N | *p* | | *q* |
| BCB composite score | (Intercept) | 0.366 | [-2.430, 3.163] | 54 | 0.827 | | 1 |
| BCB composite score | serum CRP | 0.081 | [-1.666, 1.828] | 54 | 0.939 | | 0.949 |
| BCB composite score | age | 0.046 | [0.011, 0.080] | 54 | 0.03* | | 0.039* |
| BCB composite score | sex | -0.43 | [-1.358, 0.497] | 54 | 0.44 | | 0.443 |
| BCB composite score | smoker status | 0.737 | [-0.049, 1.524] | 54 | 0.122 | | 0.21 |
| BCB composite score | BMI | -0.082 | [-0.190, 0.026] | 54 | 0.21 | | 0.21 |
| **Table S5.7 Bayesian test statistics** | | | |  |  |  |  |
| Response | Predictor | Estimate | 95% CI [LL, UL] | *BF10* | |  |  |
| BCB composite score | BC neutrophil granulocytes | 0.24 | [-0.244, 0.722] | 0.385 | |  |  |
| BCB composite score | BC monocytes | 0.211 | [-0.277, 0.699] | 0.362 | |  |  |
| BCB composite score | BC lymphocytes | 0.014 | [-0.487, 0.517] | 0.252 | |  |  |
| BCB composite score | NLR | 0.14 | [-0.343, 0.634] | 0.287 | |  |  |
| BCB composite score | MLR | 0.09 | [-0.381, 0.570] | 0.26 | |  |  |
| BCB composite score | serum CRP | -0.015 | [-0.536, 0.501] | 0.245 | |  |  |

| **Table S6 Complete parameter estimates – association analyses of cerebroventricular measures and blood-CSF barrier (BCB) composite score in SSD** | | | | | | | |
| --- | --- | --- | --- | --- | --- | --- | --- |
|  |  |  |  |  |  | |  |
| **Table S6.1 Association between BCB composite score and left choroid plexus volume** | | | | |  | |  |
| Response | Predictor | Estimate | 95% CI [LL, UL] | N | *p* | | *q* |
| Left choroid plexus | (Intercept) | 903.942 | [688.902, 1118.981] | 28 | <0.001 | | <0.001 |
| Left choroid plexus | BCB composite score | 10.038 | [-23.318, 43.393] | 28 | 0.611 | | 0.941 |
| Left choroid plexus | age | -5.64 | [-12.065, 0.785] | 28 | 0.146 | | 0.468 |
| Left choroid plexus | sex | 7.911 | [-175.489, 191.310] | 28 | 0.942 | | 0.942 |
|  |  |  |  |  |  | |  |
| **Table S6.2 Association between BCB composite score and right choroid plexus volume** | | | | |  | |  |
| Response | Predictor | Estimate | 95% CI [LL, UL] | N | *p* | | *q* |
| Right choroid plexus | (Intercept) | 819.797 | [638.423, 1001.172] | 28 | <0.001 | | <0.001 |
| Right choroid plexus | BCB composite score | 8.084 | [-20.050, 36.218] | 28 | 0.627 | | 0.941 |
| Right choroid plexus | age | -4.085 | [-9.504, 1.334] | 28 | 0.209 | | 0.468 |
| Right choroid plexus | sex | -82.558 | [-237.247, 72.130] | 28 | 0.37 | | 0.942 |
|  |  |  |  |  |  | |  |
| **Table S6.3 Association between BCB composite score and left lateral ventricle volume** | | | | |  | |  |
| Response | Predictor | Estimate | 95% CI [LL, UL] | N | *p* | | *q* |
| Left lateral ventricle | (Intercept) | 10419.46 | [6577.246, 14261.675] | 28 | <0.001 | | <0.001 |
| Left lateral ventricle | BCB composite score | -47.78 | [-643.763, 548.202] | 28 | 0.892 | | 0.984 |
| Left lateral ventricle | age | -56.766 | [-171.561, 58.029] | 28 | 0.406 | | 0.491 |
| Left lateral ventricle | sex | -1174.105 | [-4450.994, 2102.783] | 28 | 0.546 | | 0.942 |
|  |  |  |  |  |  | |  |
| **Table S6.4 Association between BCB composite score and right lateral ventricle volume** | | | | |  | |  |
| Response | Predictor | Estimate | 95% CI [LL, UL] | N | *p* | | *q* |
| Right lateral ventricle | (Intercept) | 9038.154 | [6191.923, 11884.384] | 28 | <0.001 | | <0.001 |
| Right lateral ventricle | BCB composite score | -183.642 | [-625.133, 257.849] | 28 | 0.484 | | 0.941 |
| Right lateral ventricle | age | -40.696 | [-125.734, 44.341] | 28 | 0.421 | | 0.491 |
| Right lateral ventricle | sex | -804.122 | [-3231.571, 1623.327] | 28 | 0.576 | | 0.942 |
|  |  |  |  |  |  | |  |
| **Table S6.5 Association between BCB composite score and third ventricle volume** | | | |  |  | |  |
| Response | Predictor | Estimate | 95% CI [LL, UL] | N | *p* | | *q* |
| 3rd ventricle | (Intercept) | 1230.933 | [796.736, 1665.131] | 28 | <0.001 | | <0.001 |
| 3rd ventricle | BCB composite score | 36.555 | [-30.795, 103.905] | 28 | 0.362 | | 0.941 |
| 3rd ventricle | age | -5.308 | [-18.280, 7.665] | 28 | 0.491 | | 0.491 |
| 3rd ventricle | sex | -36.182 | [-406.493, 334.13] | 28 | 0.869 | | 0.942 |
|  |  |  |  |  |  | |  |
| **Table S6.6 Association between BCB composite score and fourth ventricle volume** | | | | |  | |  |
| Response | Predictor | Estimate | 95% CI [LL, UL] | N | *p* | | *q* |
| 4th ventricle | (Intercept) | 2516.088 | [1793.669, 3238.507] | 28 | <0.001 | | <0.001 |
| 4th ventricle | BCB composite score | 1.348 | [-110.709, 113.406] | 28 | 0.984 | | 0.984 |
| 4th ventricle | age | -15.406 | [-36.990, 6.178] | 28 | 0.234 | | 0.468 |
| 4th ventricle | sex | -71.936 | [-688.061, 544.190] | 28 | 0.843 | | 0.942 |
| **Table S6.7 Bayesian test statistics** | | | |  |  |  |  |
| Response | Predictor | Estimate | 95% CI [LL, UL] | *BF10* | |  |  |
| Left choroid plexus | BCB composite score | 0.435 | [-0.292, 1.159] | 0.791 | |  |  |
| Right choroid plexus | BCB composite score | -0.072 | [-0.806, 0.663] | 0.387 | |  |  |
| Left lateral ventricle | BCB composite score | -0.063 | [-0.793, 0.678] | 0.374 | |  |  |
| Right lateral ventricle | BCB composite score | -0.262 | [-1.006, 0.484] | 0.492 | |  |  |
| 3rd ventricle | BCB composite score | 0.301 | [-0.433, 1.034] | 0.512 | |  |  |
| 4th ventricle | BCB composite score | -0.015 | [-0.759, 0.722] | 0.361 | |  |  |

| **Table S7 Complete parameter estimates – association analyses of cerebroventricular measures and blood-CSF barrier (BCB) composite score in SSD including BMI as a covariate** | | | | | | |
| --- | --- | --- | --- | --- | --- | --- |
|  |  |  |  |  |  |  |
| **Table S7.1 Association between BCB composite score and left choroid plexus volume** | | | | |  |  |
| Response | Predictor | Estimate | 95% CI [LL, UL] | N | *p* | *q* |
| Left choroid plexus | (Intercept) | 519.680 | [162.940, 876.419] | 28 | 0.020 | 0.060 |
| Left choroid plexus | BCB composite score | 19.411 | [-7.639, 46.461] | 28 | 0.231 | 0.885 |
| Left choroid plexus | age | 0.406 | [-5.183, 5.995] | 28 | 0.902 | 0.902 |
| Left choroid plexus | sex | -83.013 | [-233.810, 67.785] | 28 | 0.355 | 0.955 |
| Left choroid plexus | BMI | -2.735 | [-16.948, 11.478] | 28 | 0.745 | 0.894 |
|  |  |  |  |  |  |  |
| **Table S7.2 Association between BCB composite score and right choroid plexus volume** | | | | |  |  |
| Response | Predictor | Estimate | 95% CI [LL, UL] | N | *p* | *q* |
| Right choroid plexus | (Intercept) | 125.938 | [-281.929, 533.805] | 28 | 0.602 | 0.318 |
| Right choroid plexus | BCB composite score | 0.175 | [-30.752, 31.102] | 28 | 0.992 | 0.992 |
| Right choroid plexus | age | 1.272 | [-5.118, 7.661] | 28 | 0.736 | 0.883 |
| Right choroid plexus | sex | -32.841 | [-205.251, 139.568] | 28 | 0.747 | 0.955 |
| Right choroid plexus | BMI | 14.113 | [-2.137, 30.363] | 28 | 0.150 | 0.462 |
|  |  |  |  |  |  |  |
| **Table S7.3 Association between BCB composite score and left lateral ventricle volume** | | | | |  |  |
| Response | Predictor | Estimate | 95% CI [LL, UL] | N | *p* | *q* |
| Left lateral ventricle | (Intercept) | 5252.825 | [-2633.808, 13139.458] | 28 | 0.265 | 0.318 |
| Left lateral ventricle | BCB composite score | 28.658 | [-569.352, 626.668] | 28 | 0.935 | 0.992 |
| Left lateral ventricle | age | -93.278 | [-216.831, 30.275] | 28 | 0.209 | 0.485 |
| Left lateral ventricle | sex | -586.034 | [-3919.794, 2747.725] | 28 | 0.766 | 0.955 |
| Left lateral ventricle | BMI | 234.884 | [-79.329, 549.097] | 28 | 0.213 | 0.426 |
|  |  |  |  |  |  |  |
| **Table S7.4 Association between BCB composite score and right lateral ventricle volume** | | | | |  |  |
| Response | Predictor | Estimate | 95% CI [LL, UL] | N | *p* | *q* |
| Right lateral ventricle | (Intercept) | 4756.487 | [-1033.094, 10546.068] | 28 | 0.172 | 0.258 |
| Right lateral ventricle | BCB composite score | -120.297 | [-559.296, 318.702] | 28 | 0.643 | 0.992 |
| Right lateral ventricle | age | -70.954 | [-161.655, 19.746] | 28 | 0.193 | 0.485 |
| Right lateral ventricle | sex | -316.779 | [-2764.093, 2130.535] | 28 | 0.826 | 0.955 |
| Right lateral ventricle | BMI | 194.652 | [-36.012, 425.316] | 28 | 0.162 | 0.426 |
|  |  |  |  |  |  |  |
| **Table S7.5 Association between BCB composite score and third ventricle volume** | | | |  |  |  |
| Response | Predictor | Estimate | 95% CI [LL, UL] | N | *p* | *q* |
| 3rd ventricle | (Intercept) | 799.995 | [-105.613, 1705.602] | 28 | 0.144 | 0.258 |
| 3rd ventricle | BCB composite score | 42.931 | [-25.738, 111.599] | 28 | 0.295 | 0.885 |
| 3rd ventricle | age | -8.353 | [-22.541, 5.834] | 28 | 0.323 | 0.485 |
| 3rd ventricle | sex | 12.868 | [-369.941, 395.677] | 28 | 0.955 | 0.955 |
| 3rd ventricle | BMI | 19.591 | [-16.489, 55.672] | 28 | 0.362 | 0.543 |
|  |  |  |  |  |  |  |
| **Table S7.6 Association between BCB composite score and fourth ventricle volume** | | | | |  |  |
| Response | Predictor | Estimate | 95% CI [LL, UL] | N | *p* | *q* |
| 4th ventricle | (Intercept) | 2469.317 | [934.579, 4004.055] | 28 | 0.011 | 0.060 |
| 4th ventricle | BCB composite score | 2.040 | [-114.333, 118.413] | 28 | 0.976 | 0.992 |
| 4th ventricle | age | -15.736 | [-39.780, 8.307] | 28 | 0.274 | 0.485 |
| 4th ventricle | sex | -66.612 | [-715.362, 582.137] | 28 | 0.862 | 0.955 |
| 4th ventricle | BMI | 2.126 | [-59.020, 63.272] | 28 | 0.953 | 0.953 |

| **Table S8 Complete parameter estimates – association analyses of general disease characteristic measures and blood-CSF barrier (BCB) composite score in SZ and SZA** | | | | | | |
| --- | --- | --- | --- | --- | --- | --- |
|  |  |  |  |  |  |  |
| **Table S8.1 Association between duration of illness (DUI) and BCB composite score** | | | |  |  |  |
| Response | Predictor | Estimate | 95% CI [LL, UL] | N | *p* | *q* |
| BCB composite score | (Intercept) | -1.387 | [-2.777, 0.003] | 42 | 0.101 | 0.152 |
| BCB composite score | DUI (months) | -0.003 | [-0.008, 0.003] | 42 | 0.409 | 0.534 |
| BCB composite score | age | 0.048 | [0.002, 0.094] | 42 | 0.089 | 0.125 |
| BCB composite score | sex | -0.576 | [-1.636, 0.485] | 42 | 0.366 | 0.408 |
|  |  |  |  |  |  |  |
| **Table S8.2 Association between duration of antipsychotic treatment and BCB composite score** | | | | |  |  |
| Response | Predictor | Estimate | 95% CI [LL, UL] | N | *p* | *q* |
| BCB composite score | (Intercept) | -1.487 | [-2.895, -0.079] | 42 | 0.083 | 0.152 |
| BCB composite score | Duration AP treatment (months) | -0.002 | [-0.007, 0.003] | 42 | 0.534 | 0.534 |
| BCB composite score | age | 0.048 | [0.001, 0.094] | 42 | 0.092 | 0.125 |
| BCB composite score | sex | -0.607 | [-1.705, 0.492] | 42 | 0.357 | 0.408 |
|  |  |  |  |  |  |  |
| **Table S8.3 Association between FEP status and BCB composite score** | | | |  |  |  |
| Response | Predictor | Estimate | 95% CI [LL, UL] | N | *p* | *q* |
| BCB composite score | (Intercept) | -0.907 | [-2.294, 0.480] | 43 | 0.277 | 0.277 |
| BCB composite score | FEP status | -0.637 | [-1.489, 0.215] | 43 | 0.215 | 0.534 |
| BCB composite score | age | 0.036 | [-0.003, 0.074] | 43 | 0.125 | 0.125 |
| BCB composite score | sex | -0.514 | [-1.548, 0.520] | 43 | 0.408 | 0.408 |

| **Table S9 Complete parameter estimates – association analyses of disease severity measures and blood-CSF barrier (BCB) composite score in SZ and SZA** | | | | | | |
| --- | --- | --- | --- | --- | --- | --- |
|  |  |  |  |  |  |  |
| **Table S9.1 Association between BCB composite score and GAF score** | | | |  |  |  |
| Response | Predictor | Estimate | 95% CI [LL, UL] | N | *p* | *q* |
| GAF | (Intercept) | 38.212 | [29.161, 47.263] | 36 | <0.001*** | <0.001*** |
| GAF | BCB composite score | 0.949 | [-0.585, 2.483] | 36 | 0.303 | 0.455 |
| GAF | age | 0.262 | [-0.013, 0.538] | 36 | 0.117 | 0.282 |
| GAF | sex | -6.541 | [-12.620, -0.463] | 36 | 0.078 | 0.212 |
|  |  |  |  |  |  |  |
| **Table S9.2 Association between BCB composite score and treatment resistance** | | | | |  |  |
| Response | Predictor | Estimate | 95% CI [LL, UL] | N | *p* | *Q* |
| Clozapine lifetime | (Intercept) | -6.584 | [-14.976, -1.808] | 40 | 0.197 | 0.197 |
| Clozapine lifetime | BCB composite score | 0.678 | [0.157, 1.200] | 40 | 0.032* | 0.096 |
| Clozapine lifetime | age | 0.055 | [-0.028, 0.137] | 40 | 0.276 | 0.331 |
| Clozapine lifetime | sex | -2.375 | [-5.722, 0.972] | 40 | 0.243 | 0.243 |
| Clozapine lifetime | smoking status | 0.052 | [-1.785, 1.889] | 40 | 0.963 | 0.963 |
| Clozapine lifetime | BMI | 0.106 | [-0.177, 0.390] | 40 | 0.538 | 0.538 |
|  |  |  |  |  |  |  |
| **Table S9.3 Association between BCB composite score and PANSS total score** | | | | |  |  |
| Response | Predictor | Estimate | 95% CI [LL, UL] | N | *p* | *q* |
| PANSS total score | (Intercept) | 71.428 | [59.963, 82.893] | 36 | <0.001*** | <0.001*** |
| PANSS total score | BCB composite score | -1.826 | [-3.899, 0.248] | 36 | 0.146 | 0.292 |
| PANSS total score | age | -0.316 | [-0.669, 0.038] | 36 | 0.141 | 0.282 |
| PANSS total score | sex | 9.219 | [0.993, 17.444] | 36 | 0.067 | 0.212 |
|  |  |  |  |  |  |  |
| **Table S9.4 Association between BCB composite score and PANSS positive score** | | | | |  |  |
| Response | Predictor | Estimate | 95% CI [LL, UL] | N | *p* | *q* |
| PANSS positive score | (Intercept) | 15.229 | [11.483, 18.975] | 36 | <0.001*** | <0.001*** |
| PANSS positive score | BCB composite score | -0.024 | [-0.702, 0.654] | 36 | 0.952 | 0.952 |
| PANSS positive score | age | -0.009 | [-0.124, 0.107] | 36 | 0.899 | 0.899 |
| PANSS positive score | sex | 2.398 | [-0.290, 5.086] | 36 | 0.141 | 0.212 |
|  |  |  |  |  |  |  |
| **Table S9.5 Association between BCB composite score and PANSS negative score** | | | | |  |  |
| Response | Predictor | Estimate | 95% CI [LL, UL] | N | *p* | *q* |
| PANSS negative score | (Intercept) | 20.990 | [15.763, 26.217] | 36 | <0.001*** | <0.001*** |
| PANSS negative score | BCB composite score | -0.450 | [-1.396, 0.495] | 36 | 0.426 | 0.511 |
| PANSS negative score | age | -0.170 | [-0.331, -0.008] | 36 | 0.084 | 0.282 |
| PANSS negative score | sex | 2.848 | [0.903, 6.598] | 36 | 0.208 | 0.243 |
|  |  |  |  |  |  |  |
| **Table S9.6 Association between BCB composite score and PANSS general score** | | | | |  |  |
| Response | Predictor | Estimate | 95% CI [LL, UL] | N | *p* | *q* |
| PANSS general score | (Intercept) | 35.209 | [29.574, 40.845] | 36 | <0.001*** | <0.001*** |
| PANSS general score | BCB composite score | -1.351 | [-2.370, -0.332] | 36 | 0.032* | 0.960 |
| PANSS general score | age | -0.137 | [-0.311, 0.037] | 36 | 0.191 | 0.287 |
| PANSS general score | sex | 3.973 | [-0.070, 8.016] | 36 | 0.106 | 0.212 |

| **Table S10 Complete parameter estimates – association analyses of cognitive measures and blood-CSF barrier (BCB) composite score in SZ and SZA** | | | | | | |
| --- | --- | --- | --- | --- | --- | --- |
|  |  |  |  |  |  |  |
| **Table S10.1 Association between BCB composite score and TMT A time** | | | |  |  |  |
| Response | Predictor | Estimate | 95% CI [LL, UL] | N | *p* | *q* |
| TMT A time | (Intercept) | 39.106 | [12.797, 65.415] | 25 | 0.019* | 0.285 |
| TMT A time | BCB composite score | -1.012 | [-3.642, 1.618] | 25 | 0.515 | 0.520 |
| TMT A time | age | 0.561 | [0.019, 1.104] | 25 | 0.089 | 0.267 |
| TMT A time | sex | -3.351 | [-16.807, 10.104] | 25 | 0.672 | 0.970 |
| TMT A time | education (years) | -1.437 | [-3.067, -3.067] | 25 | 0.144 | 0.216 |
|  |  |  |  |  |  |  |
| **Table S10.2 Association between BCB composite score and TMT B time** | | | |  |  |  |
| Response | Predictor | Estimate | 95% CI [LL, UL] | N | *p* | *q* |
| TMT B time | (Intercept) | 158.223 | [22.862, 293.584] | 25 | 0.057 | 0.570 |
| TMT B time | BCB composite score | -5.134 | [-18.665, 8.397] | 25 | 0.520 | 0.520 |
| TMT B time | age | 0.495 | [-2.295, 3.284] | 25 | 0.763 | 0.763 |
| TMT B time | sex | -1.505 | [-70.735, 67.725] | 25 | 0.970 | 0.970 |
| TMT B time | education (years) | -4.870 | [-13.256, 3.516] | 25 | 0.328 | 0.328 |
|  |  |  |  |  |  |  |
| **Table S10.3 Association between BCB composite score and MoCA score** | | | |  |  |  |
| Response | Predictor | Estimate | 95% CI [LL, UL] | N | *p* | *q* |
| MoCA score | (Intercept) | 18.512 | [12.239, 24.785] | 26 | <0.001*** | <0.001*** |
| MoCA score | BCB composite score | 0.454 | [-0.173, 1.081] | 26 | 0.226 | 0.520 |
| MoCA score | age | -0.083 | [-0.212, 0.047] | 26 | 0.284 | 0.426 |
| MoCA score | sex | -2.080 | [-5.299, 1.139] | 26 | 0.279 | 0.837 |
| MoCA score | education (years) | 0.660 | [0.278, 1.042] | 26 | 0.007** | 0.021* |

| **Table S11 Complete parameter estimates – association analyses of cardiometabolic factors and blood-CSF barrier (BCB) composite score in SZ and SZA** | | | | | | |
| --- | --- | --- | --- | --- | --- | --- |
|  |  |  |  |  |  |  |
| **Table S11.1 Association between BCB composite score and total cholesterol** | | | | |  |  |
| Response | Predictor | Estimate | 95% CI [LL, UL] | N | *p* | *q* |
| BCB composite score | (Intercept) | -3.315 | [-6.438, -0.192] | 39 | 0.082 | 0.395 |
| BCB composite score | total cholesterol | 0.033 | [0.018, 0.049] | 39 | 0.001** | 0.003** |
| BCB composite score | age | 0.012 | [-0.032, 0.056] | 39 | 0.652 | 0.655 |
| BCB composite score | sex | -0.253 | [-1.219, 0.713] | 39 | 0.661 | 0.793 |
| BCB composite score | smoker status | 0.448 | [-0.358, 1.254] | 39 | 0.354 | 0.509 |
| BCB composite score | BMI | -0.128 | [-0.248, -0.009] | 39 | 0.079 | 0.236 |
|  |  |  |  |  |  |  |
| **Table S11.2 Association between BCB composite score and LDL cholesterol** | | | | |  |  |
| Response | Predictor | Estimate | 95% CI [LL, UL] | N | *P* | *q* |
| BCB composite score | (Intercept) | -0.833 | [-3.848, 2.182] | 39 | 0.643 | 0.643 |
| BCB composite score | LDL cholesterol | 0.026 | [0.013, 0.039] | 39 | 0.002** | 0.004** |
| BCB composite score | age | 0.012 | [-0.033, 0.058] | 39 | 0.655 | 0.655 |
| BCB composite score | sex | -0.115 | [-1.117, 0.886] | 39 | 0.847 | 0.847 |
| BCB composite score | smoker status | 0.612 | [-0.210, 1.435] | 39 | 0.216 | 0.509 |
| BCB composite score | BMI | -0.107 | [-0.228, 0.013] | 39 | 0.139 | 0.236 |
|  |  |  |  |  |  |  |
| **Table S11.3 Association between BCB composite score and HDL cholesterol** | | | | |  |  |
| Response | Predictor | Estimate | 95% CI [LL, UL] | N | *p* | *q* |
| BCB composite score | (Intercept) | 3.097 | [-0.774, 6.967] | 39 | 0.185 | 0.395 |
| BCB composite score | HDL cholesterol | -0.044 | [-0.070, -0.019] | 39 | 0.005** | 0.008** |
| BCB composite score | age | 0.061 | [0.018, 0.104] | 39 | 0.023* | 0.069 |
| BCB composite score | sex | -0.326 | [-1.343, 0.691] | 39 | 0.591 | 0.793 |
| BCB composite score | smoker status | 0.410 | [-0.446, 1.266] | 39 | 0.424 | 0.509 |
| BCB composite score | BMI | -0.106 | [-0.231, 0.018] | 39 | 0.157 | 0.236 |
|  |  |  |  |  |  |  |
| **Table S11.4 Association between BCB composite score and triglycerides** | | | | |  |  |
| Response | Predictor | Estimate | 95% CI [LL, UL] | N | *P* | *q* |
| BCB composite score | (Intercept) | 1.736 | [-1.191, 4.662] | 39 | 0.323 | 0.395 |
| BCB composite score | Triglycerides | 0.013 | [0.008, 0.017] | 39 | <0.001*** | <0.001*** |
| BCB composite score | age | 0.058 | [0.021, 0.096] | 39 | 0.013* | 0.069 |
| BCB composite score | sex | -0.257 | [-1.157, 0.643] | 39 | 0.632 | 0.793 |
| BCB composite score | smoker status | 0.285 | [-0.475, 1.045] | 39 | 0.530 | 0.530 |
| BCB composite score | BMI | -0.199 | [-0.320, -0.079] | 39 | 0.009** | 0.054 |
|  |  |  |  |  |  |  |
| **Table S11.5 Association between BCB composite score and systolic blood pressure** | | | | |  |  |
| Response | Predictor | Estimate | 95% CI [LL, UL] | N | *p* | *q* |
| BCB composite score | (Intercept) | -3.504 | [-9.491, 2.482] | 41 | 0.329 | 0.395 |
| BCB composite score | systolic BP | 0.023 | [-0.025, 0.070] | 41 | 0.428 | 0.428 |
| BCB composite score | age | 0.034 | [-0.012, 0.080] | 41 | 0.219 | 0.329 |
| BCB composite score | sex | -0.385 | [-1.542, 0.772] | 41 | 0.578 | 0.793 |
| BCB composite score | smoker status | 0.487 | [-0.428, 1.402] | 41 | 0.375 | 0.509 |
| BCB composite score | BMI | -0.023 | [-0.148, 0.101] | 41 | 0.754 | 0.754 |
|  |  |  |  |  |  |  |
| **Table S11.6 Association between BCB composite score and HbA1c** | | | |  |  |  |
| Response | Predictor | Estimate | 95% CI [LL, UL] | N | *p* | *q* |
| BCB composite score | (Intercept) | -3.490 | [-8.938, 1.957] | 38 | 0.286 | 0.395 |
| BCB composite score | HbA1c | 0.581 | [-0.508, 1.671] | 38 | 0.373 | 0.428 |
| BCB composite score | age | 0.045 | [-0.006, 0.096] | 38 | 0.144 | 0.288 |
| BCB composite score | sex | -0.626 | [-1.771, 0.520] | 38 | 0.362 | 0.793 |
| BCB composite score | smoker status | 0.659 | [-0.306, 1.624] | 38 | 0.256 | 0.509 |
| BCB composite score | BMI | -0.048 | [-0.185, 0.089] | 38 | 0.556 | 0.667 |

| **Table S12 Complete parameter estimates – association analyses of peripheral inflammatory markers and blood-CSF barrier (BCB) composite score in SZ and SZA** | | | | | | |
| --- | --- | --- | --- | --- | --- | --- |
|  |  |  |  |  |  |  |
| **Table S12.1 Association between BCB composite score and absolute neutrophil count** | | | | |  |  |
| Response | Predictor | Estimate | 95% CI [LL, UL] | N | *p* | *q* |
| BCB composite score | (Intercept) | -0.804 | [-4.279, 2.670] | 40 | 0.698 | 0.751 |
| BCB composite score | BC neutrophil granulocytes | 0.050 | [-0.223, 0.322] | 40 | 0.760 | 0.922 |
| BCB composite score | age | 0.040 | [-0.004, 0.085] | 40 | 0.136 | 0.164 |
| BCB composite score | sex | -0.609 | [-1.753, 0.535] | 40 | 0.374 | 0.442 |
| BCB composite score | smoker status | 0.505 | [-0.450, 1.460] | 40 | 0.377 | 0.486 |
| BCB composite score | BMI | -0.035 | [-0.172, 0.102] | 40 | 0.669 | 0.795 |
|  |  |  |  |  |  |  |
| **Table S12.2 Association between BCB composite score and absolute monocyte count** | | | | |  |  |
| Response | Predictor | Estimate | 95% CI [LL, UL] | N | *p* | *q* |
| BCB composite score | (Intercept) | -1.082 | [-4.636, 2.472] | 40 | 0.610 | 0.751 |
| BCB composite score | BC monocytes | 1.027 | [-1.909, 3.963] | 40 | 0.558 | 0.922 |
| BCB composite score | age | 0.039 | [-0.006, 0.084] | 40 | 0.148 | 0.164 |
| BCB composite score | sex | -0.607 | [-1.735, 0.522] | 40 | 0.370 | 0.442 |
| BCB composite score | smoker status | 0.415 | [-0.583, 1.414] | 40 | 0.486 | 0.486 |
| BCB composite score | BMI | -0.035 | [-0.167, 0.098] | 40 | 0.663 | 0.795 |
|  |  |  |  |  |  |  |
| **Table S12.3 Association between BCB composite score and absolute lymphocyte count** | | | | |  |  |
| Response | Predictor | Estimate | 95% CI [LL, UL] | N | *p* | *q* |
| BCB composite score | (Intercept) | -0.803 | [-4.279, 2.673] | 40 | 0.699 | 0.751 |
| BCB composite score | BC lymphocytes | 0.159 | [-0.932, 1.250] | 40 | 0.806 | 0.922 |
| BCB composite score | age | 0.043 | [-0.005, 0.090] | 40 | 0.141 | 0.164 |
| BCB composite score | sex | -0.607 | [-1.757, 0.543] | 40 | 0.378 | 0.442 |
| BCB composite score | smoker status | 0.475 | [-0.560, 1.510] | 40 | 0.443 | 0.486 |
| BCB composite score | BMI | -0.041 | [-0.198, 0.117] | 40 | 0.665 | 0.795 |
|  |  |  |  |  |  |  |
| **Table S12.4 Association between BCB composite score and NLR** | | |  |  |  |  |
| Response | Predictor | Estimate | 95% CI [LL, UL] | N | *p* | *q* |
| BCB composite score | (Intercept) | -0.715 | [-4.490, 3.061] | 40 | 0.751 | 0.751 |
| BCB composite score | NLR | -0.032 | [-0.573, 0.510] | 40 | 0.922 | 0.922 |
| BCB composite score | age | 0.041 | [-0.005, 0.086] | 40 | 0.140 | 0.164 |
| BCB composite score | sex | -0.574 | [-1.705, 0.557] | 40 | 0.397 | 0.442 |
| BCB composite score | smoker status | 0.534 | [-0.409, 1.477] | 40 | 0.345 | 0.486 |
| BCB composite score | BMI | -0.029 | [-0.162, 0.104] | 40 | 0.714 | 0.795 |
|  |  |  |  |  |  |  |
| **Table S12.5 Association between BCB composite score and MLR** | | |  |  |  |  |
| Response | Predictor | Estimate | 95% CI [LL, UL] | N | *p* | *q* |
| BCB composite score | (Intercept) | -0.997 | [-5.292, 3.298] | 40 | 0.697 | 0.751 |
| BCB composite score | MLR | 0.421 | [-4.966, 5.808] | 40 | 0.896 | 0.922 |
| BCB composite score | age | 0.039 | [-0.007, 0.086] | 40 | 0.164 | 0.164 |
| BCB composite score | sex | -0.568 | [-1.702, 0.567] | 40 | 0.404 | 0.442 |
| BCB composite score | smoker status | 0.535 | [-0.405, 1.474] | 40 | 0.343 | 0.486 |
| BCB composite score | BMI | -0.024 | [-0.165, 0.117] | 40 | 0.773 | 0.795 |
|  |  |  |  |  |  |  |
| **Table S12.6 Association between BCB composite score and CRP** | | |  |  |  |  |
| Response | Predictor | Estimate | 95% CI [LL, UL] | N | *p* | *q* |
| BCB composite score | (Intercept) | -0.927 | [-4.637, 2.782] | 40 | 0.675 | 0.751 |
| BCB composite score | serum CRP | -0.190 | [-2.118, 1.739] | 40 | 0.869 | 0.922 |
| BCB composite score | age | 0.041 | [-0.004, 0.086] | 40 | 0.137 | 0.164 |
| BCB composite score | sex | -0.542 | [-1.721, 0.637] | 40 | 0.442 | 0.442 |
| BCB composite score | smoker status | 0.516 | [-0.450, 1.483] | 40 | 0.373 | 0.486 |
| BCB composite score | BMI | -0.022 | [-0.167, 0.122] | 40 | 0.795 | 0.795 |

| **Table S13 Complete parameter estimates – association analyses of cerebroventricular measures and blood-CSF barrier (BCB) composite score in SZ and SZA** | | | | | | |
| --- | --- | --- | --- | --- | --- | --- |
|  |  |  |  |  |  |  |
| **Table S13.1 Association between BCB composite score and left choroid plexus volume** | | | | |  |  |
| Response | Predictor | Estimate | 95% CI [LL, UL] | N | *p* | *q* |
| Left choroid plexus | (Intercept) | 513.751 | [61.369, 966.134] | 25 | 0.064 | 0.210 |
| Left choroid plexus | BCB composite score | 15.344 | [-15.784, 46.472] | 25 | 0.405 | 0.961 |
| Left choroid plexus | age | 1.145 | [-5.507, 7.797] | 25 | 0.770 | 0.770 |
| Left choroid plexus | sex | -93.014 | [-258.176, 72.149] | 25 | 0.343 | 0.874 |
| Left choroid plexus | BMI | -3.164 | [-20.343, 14.014] | 25 | 0.754 | 0.853 |
|  |  |  |  |  |  |  |
| **Table S13.2 Association between BCB composite score and right choroid plexus volume** | | | | |  |  |
| Response | Predictor | Estimate | 95% CI [LL, UL] | N | *p* | *q* |
| Right choroid plexus | (Intercept) | 65.496 | [-452.977, 583.970] | 25 | 0.830 | 0.830 |
| Right choroid plexus | BCB composite score | -1.026 | [-36.702, 34.650] | 25 | 0.961 | 0.961 |
| Right choroid plexus | age | 2.767 | [-4.857, 10.391] | 25 | 0.538 | 0.744 |
| Right choroid plexus | sex | -34.396 | [-223.689, 154.896] | 25 | 0.757 | 0.874 |
| Right choroid plexus | BMI | 14.346 | [-5.342, 34.034] | 25 | 0.223 | 0.506 |
|  |  |  |  |  |  |  |
| **Table S13.3 Association between BCB composite score and left lateral ventricle volume** | | | | |  |  |
| Response | Predictor | Estimate | 95% CI [LL, UL] | N | *p* | *q* |
| Left lateral ventricle | (Intercept) | 6368.084 | [-3453.611, 16189.779] | 25 | 0.277 | 0.416 |
| Left lateral ventricle | BCB composite score | 45.465 | [-630.360, 721.290] | 25 | 0.909 | 0.961 |
| Left lateral ventricle | age | -135.188 | [-279.612, 9.236] | 25 | 0.122 | 0.624 |
| Left lateral ventricle | sex | -440.252 | [-4026.103, 3145.600] | 25 | 0.834 | 0.874 |
| Left lateral ventricle | BMI | 247.406 | [-125.552, 620.364] | 25 | 0.266 | 0.506 |
|  |  |  |  |  |  |  |
| **Table S13.4 Association between BCB composite score and right lateral ventricle volume** | | | | |  |  |
| Response | Predictor | Estimate | 95% CI [LL, UL] | N | *p* | *q* |
| Right lateral ventricle | (Intercept) | 6111.450 | [-1341.367, 13564.267] | 25 | 0.173 | 0.346 |
| Right lateral ventricle | BCB composite score | -48.018 | [-560.842, 464.806] | 25 | 0.873 | 0.961 |
| Right lateral ventricle | age | -82.587 | [-192.178, 27.004] | 25 | 0.208 | 0.624 |
| Right lateral ventricle | sex | -416.191 | [-3137.176, 2304.795] | 25 | 0.795 | 0.874 |
| Right lateral ventricle | BMI | 161.566 | [-121.439, 444.571] | 25 | 0.337 | 0.506 |
|  |  |  |  |  |  |  |
| **Table S13.5 Association between BCB composite score and third ventricle volume** | | | |  |  |  |
| Response | Predictor | Estimate | 95% CI [LL, UL] | N | *p* | *q* |
| 3rd ventricle | (Intercept) | 362.106 | [-752.496, 1476.709] | 25 | 0.581 | 0.697 |
| 3rd ventricle | BCB composite score | 15.954 | [-60.742, 92.649] | 25 | 0.724 | 0.961 |
| 3rd ventricle | age | -4.781 | [-21.171, 11.609] | 25 | 0.620 | 0.744 |
| 3rd ventricle | sex | 42.261 | [-364.674, 449.197] | 25 | 0.860 | 0.874 |
| 3rd ventricle | BMI | 30.710 | [-11.615, 73.035] | 25 | 0.225 | 0.506 |
|  |  |  |  |  |  |  |
| **Table S13.6 Association between BCB composite score and fourth ventricle volume** | | | | |  |  |
| Response | Predictor | Estimate | 95% CI [LL, UL] | N | *p* | *q* |
| 4th ventricle | (Intercept) | 2174.137 | [212.049, 4136.225] | 25 | 0.070 | 0.210 |
| 4th ventricle | BCB composite score | -21.422 | [-156.432, 113.588] | 25 | 0.787 | 0.961 |
| 4th ventricle | age | -11.739 | [-40.591, 17.113] | 25 | 0.491 | 0.744 |
| 4th ventricle | sex | -66.839 | [-783.187, 649.510] | 25 | 0.874 | 0.874 |
| 4th ventricle | BMI | 8.098 | [-66.408, 82.604] | 25 | 0.853 | 0.853 |
